# Long-term trends in yield variance of temperate managed grassland

**DOI:** 10.1007/s13593-023-00885-w

**Published:** 2023-04-26

**Authors:** Janna Macholdt, Steffen Hadasch, Andrew Macdonald, Sarah Perryman, Hans-Peter Piepho, Tony Scott, Merete Elisabeth Styczen, Jonathan Storkey

**Affiliations:** 1 Professorship of Agronomy, Institute of Agriculture and Nutritional Sciences, Martin-Luther-University Halle-Wittenberg, Betty-Heimann-Strasse 5, 06120 Halle (Saale), Germany; 2 Biostatistics Unit, Institute of Crop Science, University of Hohenheim, Fruwirthstrasse 23, 70599 Stuttgart, Germany; 3 Protecting Crops and Environment, Rothamsted Research, Harpenden, AL5 2JQ Hertfordshire UK; 4 Computational and Analytical Sciences Department, Rothamsted Research, Hertfordshire AL5 2JQ Harpenden, UK; 5 Section of Environmental Chemistry and Physics, Department of Plant and Environmental Sciences, University of Copenhagen, Thorvaldsensvej 40, 1871 Copenhagen, Denmark

**Keywords:** Agronomic management, Biomass production, Climate resilience, Fertilizer input, Food security, Liming, Plant species diversity, Soil pH, Temperature, Water stress

## Abstract

The management of climate-resilient grassland systems is important for stable livestock fodder production. In the face of climate change, maintaining productivity while minimizing yield variance of grassland systems is increasingly challenging. To achieve climate-resilient and stable productivity of grasslands, a better understanding of the climatic drivers of long-term trends in yield variance and its dependence on agronomic inputs is required. Based on the Park Grass Experiment at Rothamsted (UK), we report for the first time the long-term trends in yield variance of grassland (1965–2018) in plots given different fertilizer and lime applications, with contrasting productivity and plant species diversity. We implemented a statistical model that allowed yield variance to be determined independently of yield level. Environmental abiotic covariates were included in a novel criss-cross regression approach to determine climatic drivers of yield variance and its dependence on agronomic management. Our findings highlight that sufficient liming and moderate fertilization can reduce yield variance while maintaining productivity and limiting loss of plant species diversity. Plots receiving the highest rate of nitrogen fertilizer or farmyard manure had the highest yield but were also more responsive to environmental variability and had less plant species diversity. We identified the days of water stress from March to October and temperature from July to August as the two main climatic drivers, explaining approximately one-third of the observed yield variance. These drivers helped explain consistent unimodal trends in yield variance—with a peak in approximately 1995, after which variance declined. Here, for the first time, we provide a novel statistical framework and a unique long-term dataset for understanding the trends in yield variance of managed grassland. The application of the criss-cross regression approach in other long-term agro-ecological trials could help identify climatic drivers of production risk and to derive agronomic strategies for improving the climate resilience of cropping systems.

**Supplementary Information:**

The online version contains supplementary material available at 10.1007/s13593-023-00885-w.

## Introduction

The management of climate-resilient grassland systems is important for stable livestock fodder production over time (Schmidhuber and Tubiello 2007; Arata et al. 2020; Trnka et al. 2021; Bengtsson et al. 2019; Bommarco et al. 2013; Reckling et al. 2021). However, in the face of climate change and the associated increases in abiotic stresses, maintaining productivity while minimizing temporal yield variance (or rather improving yield stability) of grassland systems will become increasingly challenging (Olesen and Bindi 2002; Ray et al. 2019). Observed climatic changes, such as increasing temperatures or weather anomalies, have negatively affected global grassland productivity (Kipling et al. 2016; Höglind et al. 2013; Addy et al. 2022; Brookshire and Weaver 2015; Wilcox et al. 2017; Hall and Scurlock 1991). For northern Europe as the experimental region of this study, Trnka et al. (2021) reported that the annual average temperature as well as the frequency of combined heat and drought stress for grassland has increased in recent decades. In addition, they showed that by 2050, the area of grassland exposed to combined heat and drought may double compared with that in 2021 and that northern regions will experience higher temperatures and drought more frequently (Trnka et al. 2021). As well as long-term temporal trends, these changes in climatic conditions are expected to increase the interannual yield variance of grassland systems and decrease plant species diversity, which could put future food security at risk (Graux et al. 2013; Piseddu et al. 2021).

The intensification of grassland production through the use of inorganic fertilizers and simplified swards of fast-growing grass cultivars has delivered increased primary biomass and live weight gain of livestock (Carswell et al. 2019). However, the use of inorganic fertilizer is a major contributor to greenhouse gas emissions (both in manufacturing and application) and results in swards with low plant species diversity that may also compromise resilience (Robertson and Vitousek 2009; Storkey et al. 2015). Recent findings of short-term grassland studies have confirmed that additional nutrient inputs often increase not only yield but also interannual yield variance and reduce plant species diversity (Hautier et al. 2020, 2014; Zhang et al. 2016, 2019; Crawley et al. 2005). Moreover, studies reported that relative climatic adaptability decreased with increasing land use intensity (Deguines et al. 2014). In particular, mineral N (nitrogen) fertilizer input reduces the diversity of terrestrial vegetation by favouring fast-growing grass species adapted to high nutrient availability (Midolo et al. 2018). There is evidence that greater plant species diversity in managed grasslands may enhance their resilience to climate change and result in more stable yields in response to disturbance (Tracy and Sanderson 2004; Tilman et al. 2006; Haughey et al. 2018; Sanderson 2010; Baca Cabrera et al. 2021). However, the relationship between stable productivity (low interannual yield variance) and plant species diversity can also strongly vary depending on agronomic management and soil conditions (Bullock et al. 2001; Hector et al. 1999; Tracy and Sanderson 2004; Crawley et al. 2005; Storkey et al. 2015). A very important agronomic management factor for stabilizing yields over time and for maintaining plant species diversity is liming, particularly in soils prone to acidification (Fornara et al. 2011; Storkey et al. 2015).

To achieve resilient, sustainable, and stable productivity of grasslands, a better understanding of the climatic drivers of long-term trends in temporal yield variance and its dependence on agronomic inputs and biological diversity is required. Ideally, such assessments should be done on long-term datasets (>20 years) (Piepho 1998; Dodd et al. 1997) because estimates of yield variance based on a shorter period might be imprecise and long-term trends cannot be detected (Hadasch et al. 2020; Macholdt et al. 2021; Reckling et al. 2021). Data over decadal time scales also allow the adaptation of grasslands to climatic trends (such as increasing temperatures) to be quantified in addition to the buffering of short-term climatic variability. Long-term experiments (LTEs) and their associated datasets provide a unique opportunity to examine the effects of climate change (Berti et al. 2016).

Appropriate statistical methods are necessary to handle the often complex design of LTEs and any experimental modifications that have occurred over time (Reckling et al. 2021; Payne 2015; Macdonald et al. 2018), such as those used for genotype × environment × management interaction analyses in other disciplines, including plant breeding (Hadasch et al. 2020) and agronomy (Macholdt et al. 2021). Although there are some recent studies on the yield variance of field crops, detailed knowledge about the long-term effects of climatic changes and agronomic management on temporal trends in yield variance for grasslands is limited (Dodd et al. 1997). Previous studies reporting yield variance of grasslands often cover only short periods with less than 10 years (Tilman et al. 2006; Prieto et al. 2015; Hautier et al. 2014, 2020; Haughey et al. 2018; Zhang et al. 2016. 2019; Sanderson 2010; Dodd et al. 1997). To date, there have been only few analyses of permanent, managed grassland systems that include temporal trends in yield variance covering a long-term period (Craven et al. 2018; Isbell et al. 2017; Dodd et al. 1997). These studies often neglect a further requirement, which is that yield variance should be determined independently of yield level, otherwise yield variance can be incorrectly interpreted if the time span is too short and there is a systematic dependency of variation on the mean (Preissel et al. 2015).

In this study, we used a long-term dataset (1965–2018) from The Park Grass Experiment at Rothamsted (UK) with consistent long-term management and determined yield variance independently of yield level, for most accurate statistical estimates. The novelty of our analysis is that we included environmental abiotic covariates, such as atmospheric chemistry (wet and dry N deposition, SO_2_, CO_2_) and other climatic parameters (air temperature, precipitation, soil moisture deficit, etc.), in a novel criss-cross regression approach (extended Finlay–Wilkinson regression) to determine the main climatic drivers of long-term yield variance and to evaluate the effects of liming and fertilizer applications on the relative impact of environmental variability on yield sensitivity (or responsiveness) across a range of plots with contrasting productivity and plant species richness.

We specifically addressed the following three research questions:

I. Are there temporal trends in interannual yield variance over the study period, and do these vary in relation to fertilizer and lime applications?
II. What are the most important environmental abiotic drivers explaining yield variance, and does related yield sensitivity to these climatic drivers depend on agronomic management?
III. Is there a correlation between plant species diversity, mean yield, and yield variance?

## Material and methods

### The Park Grass Experiment (PGE)

**Fig. 1:**
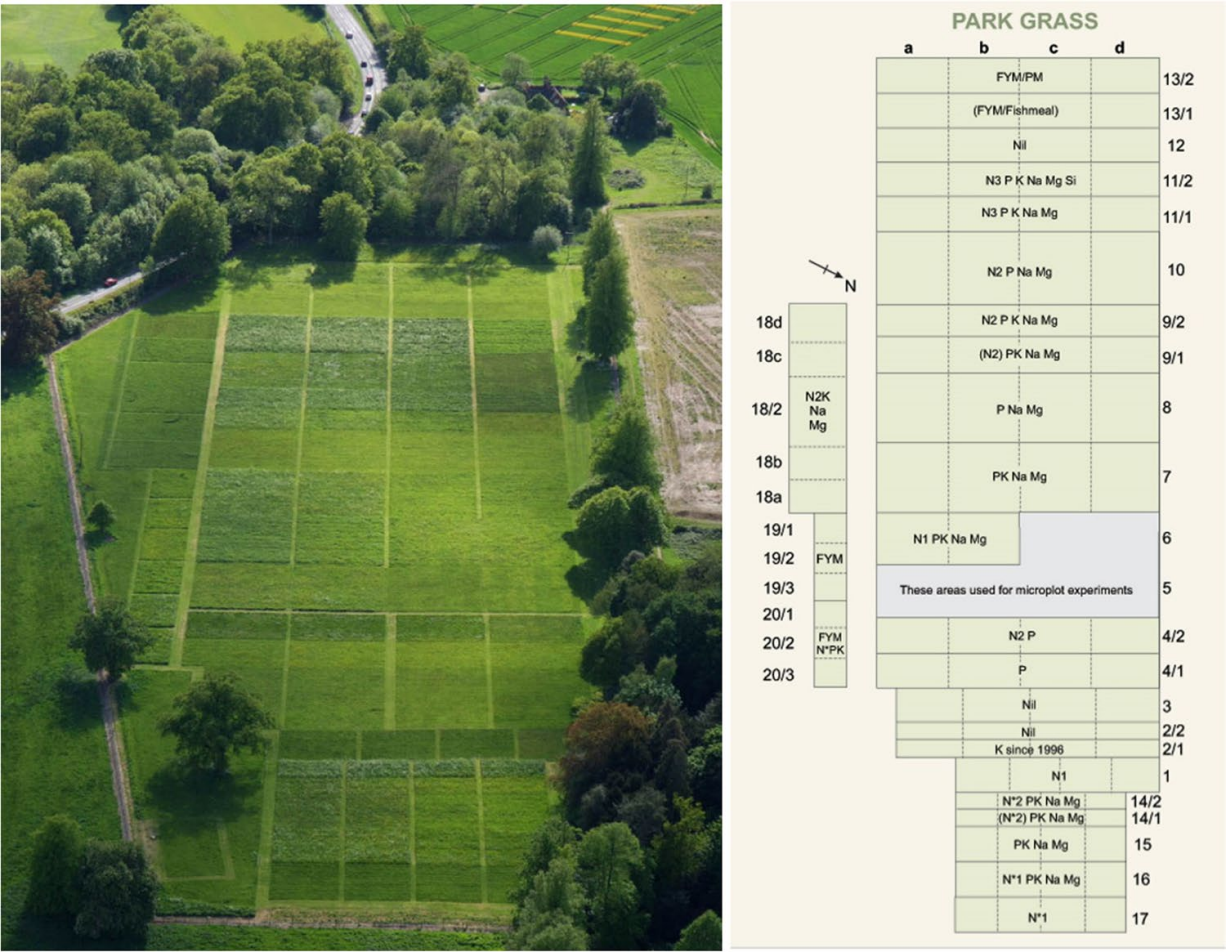
Park Grass Experiment aerial view (left) and plot layout (right). Location: Harpenden, UK, Herts, AL5 2JQ (51°48′12.33″N; 0°22′21.66″W; 130 m a.s.l.). Detailed information about plot layout and treatments are shown in Tables A1 and A3 Supplementary material. Source: electronic Rothamsted Archive (http://www.era.rothamsted.ac.uk/Park#images/).

This study was based on the Park Grass Experiment (PGE) at Rothamsted (Fig. 1), initiated by Lawes and Gilbert in 1856 to examine the effects of different mineral fertilizers and organic manures on the productivity of permanent pasture cut for hay (Silvertown et al. 2006). A detailed description of the experiment is available in the Rothamsted Guide to Classical Experiments (Macdonald et al. 2018) and the e-RA website (http://www.era.rothamsted.ac.uk/experiment/rpg5). The Park Grass soil is a moderately well-drained silty clay loam overlying clay-with-flints (Avery and Catt 1995), a Chromic or Vertic Luvisol according to the FAO classification. The experimental site shows a relatively uniform soil, based on comprehensive soil analyses made by Avery and Catt (1995)—the ‘Soils at Rothamsted Colour Map’ is provided in Fig. A1-b Supplementary material and available online (https://doi.org/10.23637/ERADOC-1-143; p. 43). In 1856, starting values for PGE soil parameters in the topsoil (0–23 cm) were estimated as pH 5.7, 11.6% sand, 66.3% silt, 22.1% clay, soil weight 2430 t ha^−1^, bulk density 1.1 g cm^3^, and total soil nitrogen content of 5830 kg N ha^−1^ (Lawes and Gilbert 1859).

The mean annual air temperature and rainfall at the Rothamsted site (1981–2010) were 9.8 °C and 733 mm, respectively (Perryman et al. 2019). In recent decades, the annual mean air temperature (1989–2018) was 1.1 °C warmer compared to the previous period (1878–1988: 9.04 °C) (Macdonald et al. 2018). The increasing trend in temperature anomaly at Rothamsted (1880–2018) is provided in Fig. A2 Supplementary material and available online (https://doi.org/10.23637/rms-RMAAtempanomaly-1). The effective plant available water capacity of 135 mm in the effective root zone of the Park Grass site implies that plant growth is often limited by lack of water in summer (Avery and Catt 1995).

The current analysis is focused on the period from 1965 to 2018 using plots with constant treatments and a consistent harvesting methodology to ensure comparability and accurate estimates of temporal trends in yield variance. The design of the PGE is provided in Fig. 1 (more details are provided in Fig. A1-a Supplementary material), consisting of 24 main plots with contrasting fertilizer treatments, each divided into four sub-plots ‘a, b, c, and d’ for liming treatments. Sub-plots a, b, and c receive lime, if needed, every 3 years to maintain soil pH 7, 6, and 5, respectively; sub-plot d receives no chalk. The yield data selected for this study were taken from the a, b, c, and d sub-plots of seven contrasting main plots (red marked in Fig. A1 Supplementary material) chosen to represent a range of productivity and species richness (*plot3*: Nil/unfertilized, *plot7/2*: ‘P K Na Mg’, *plot6*: ‘N1 P K Na Mg’ with 48 kg N ha^−1^ ammonium sulfate, *plot 9/2*: ‘N2 P K Na Mg’ with 96 kg N ha^−1^ ammonium sulfate, *plot11/1*: ‘N3 P K Na Mg Si’ with 144 kg N ha^−1^ ammonium sulfate, *plot13/2*: manure applied in a 4-year cycle, *plot17*: N*1 with 48 kg N ha^−1^ sodium nitrate), except on plot 6, where only the a and b sub-plots were available. The plots 7/2, 6, 9/2, 11/1, and 17 represent different levels and forms of inorganic fertilization, whereas the plot 13/2 represents organic fertilization (poultry manure since 2003, before farmyard manure). Additional treatment details are provided in Table A3 Supplementary material. Yield data were obtained from the electronic Rothamsted Archive ‘e-RA’ and have been made publicly available on the e-RA website (https://doi.org/10.23637/rpg5-yields1965-2018-01) (Perryman and Ostler 2021) and included plot-specific twice-yearly yields (total aboveground biomass; sum of 1st and 2nd cuts; 100% dry matter). The first cut was made into hay and removed in mid-June, and the second cut was taken with a forage harvester while still green (end-October, biomass removed).

The plant communities on the PGE are naturally assembled from a regional species pool that is classified as dicotyledon-rich *Cynosurus cristatus–Centaurea nigra* grassland, one of the mesotrophic grassland communities in the British National Vegetation Classification system (Dodd et al. 1994). The botanical composition of all sub-plots was studied annually from 1991 to 2000 and from 2010 to 2012 by recording the dry mass of each plant species in early June (number of species, Shannon’s diversity index) (Storkey et al. 2015; Crawley et al. 2005). The species of grasses, forbs, and legumes comprising at least 5% of the aboveground biomass found in these surveys and used for this study have been made publicly available on the e-RA website (https://doi.org/10.23637/rpg5-species_1991-2000-01) (Perryman et al. 2021). An overview about the changes in the number of plant species over time is provided in Fig. A11 Supplementary material; a more detailed description of the PGE biodiversity data has been reported in the Rothamsted Guide to Classical Experiments on pp. 25–27 (Macdonald et al. 2018) and is available online: http://www.era.rothamsted.ac.uk/home/Web_LTE_Guidebook_2018_2019-reprint.pdf. Because of the resource required for vegetation assessments, data on plant diversity are only available for a sub-set of the years and plots used in this analysis. However, the data cover a significant proportion of the time period analysed in our study that includes large interannual variation in weather and yields and a period of change in atmospheric chemistry (Crawley et al. 2005; Storkey et al. 2015; Ray et al. 2015). Although plant communities respond to these drivers, the relative differences between the plots in species richness and diversity are conserved in time (dynamic equilibrium); this meant that the relationship between plant diversity and yield variance could be included in our analysis (Silvertown et al. 2006).

### Environmental abiotic covariates

**Fig. 2:**
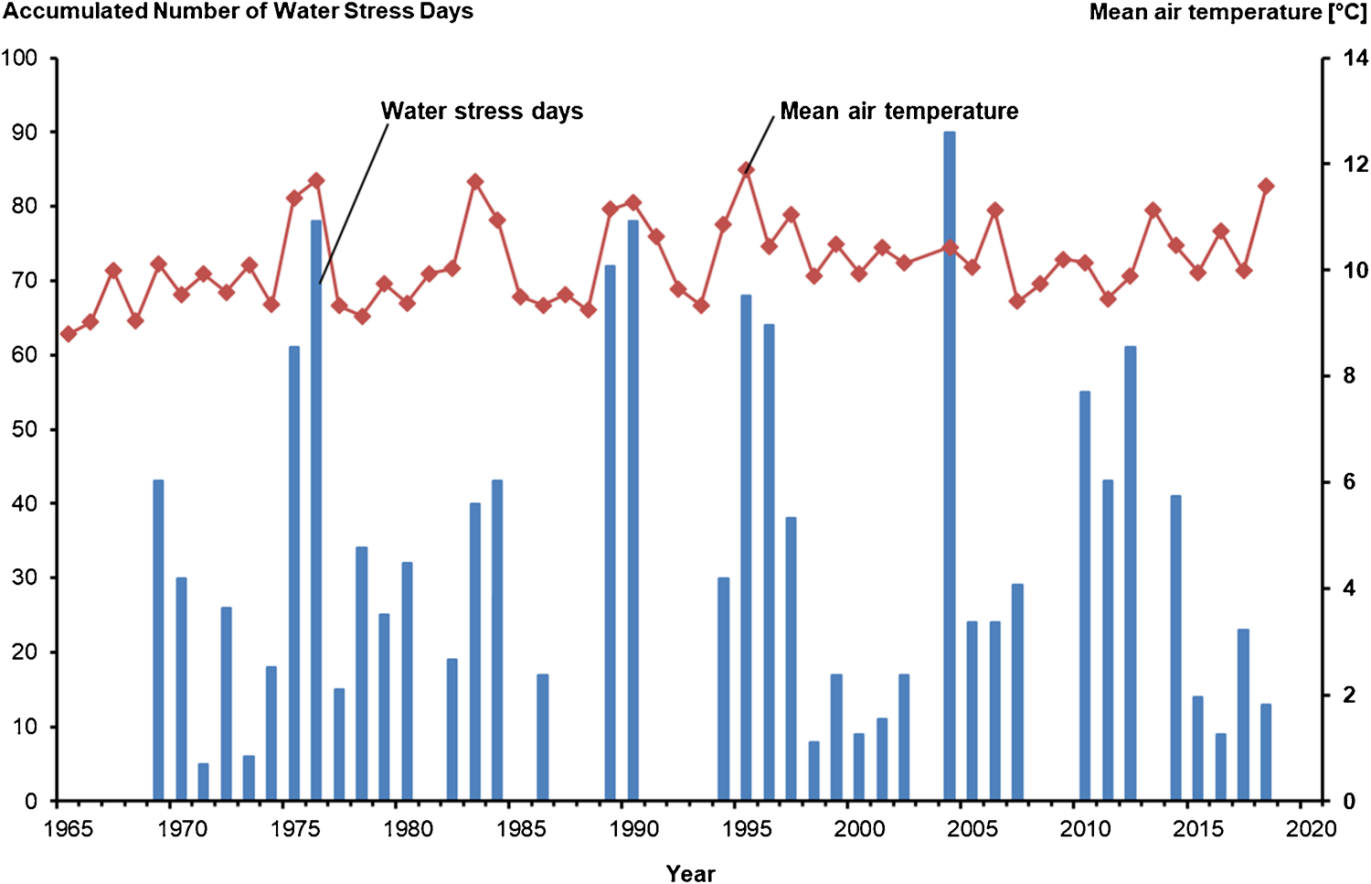
Accumulated number of water stress days for the vegetation period from March to October and temporal development of the mean air temperature (°C) for the months July–August at Rothamsted (1965–2018). Water stress was defined as a limited plant available soil water content (formula described in “Material and methods” section). Further information about the temperature anomaly is provided in Fig. A2 Supplementary material.

Based on statistical analyses (see Section 2.3), the effect of the following environmental abiotic covariates on temporal yield variance was tested. Different climatic covariables (air temperature, humidity, rainfall, hours of sunshine, soil moisture, radiation, wind) were measured daily at Rothamsted Research (available at: http://www.era.rothamsted.ac.uk/#measurements), except for measurements of atmospheric carbon dioxide concentration (Fig. A4 Supplementary material) that was recorded monthly. In addition, air chemistry parameters were measured, N deposition, and SO_2_emissions (1965–2018, UK), which are shown in Fig. A5 Supplementary material. Besides these measured covariates, the accumulated numbers of water stress days for the vegetation periods 1st March–15th of June (time period up to typical date for 1st cut/harvest), 16th June–31st October (time period up to typical date for 2nd cut/harvest), and for the entire period (Fig. 2) were calculated yearly in the following manner. The amount of maximum plant available water (MPAW [mm]), which acted as a ‘bucket of water’, was specified for the soil. At the start of the calculation (1 Jan 1965), it is assumed that the actual plant available water (APAW(t)) equaled the MPAW (‘the bucket was full’). The daily water surplus was calculated as rainfall minus potential evaporation over grass (WS(t)), with potential evaporation over grass as a derived meteorological variable (see formula details: http://www.era.rothamsted.ac.uk/info/met/derived_variables#EVAPG). A daily balance was calculated as DB(t) = APAW(t − 1) + WS(t). For positive WS(t)-values, APAW increased to the maximum of MPAW. Above this value, water was assumed to run off or drain away. For negative values, APAW was reduced, and when reaching 0, no more water extraction was possible.

- If DB(t) > MPAW; APAW(t) = MPAW
- If MPAW > DB(t) > 0; APAW(t) = DB(t)
- If DB(t) < 0; APAW(t) = 0

Days where APAW was 0 were counted as stress days. The numbers of water stress days accumulated for the 1st March–15th of June (harvest time of the 1st cut), 16th June–31st of October (harvest time of the 2nd cut), and for the entire period each year were determined. No water stress occurred before March or after October. Several values of MPAW were tested, but an MPAW of 135 mm provided the best correlation for both the 2nd cut and the yearly yield, in line with the soil description (Kohler et al. 2016).

### Statistical analysis

To account for the experimental design, a mixed model was used based on REML, as recommended by Raman et al. (Raman et al. 2011) and Onofri et al. (Onofri et al. 2016). Each plot had a different ‘fertilizer × liming’ treatment combination, with no replication or randomization (Fig. A1 Supplementary material), so that plot errors and ‘fertilizer × liming’ interactions (=residual) could not be separated. The size of the main plots (306–1912 m^2^) might partly compensate for the lack of replication, particularly because the experimental site was reasonably uniform when the experiment started in 1856 (Crawley et al. 2005; Lawes and Gilbert 1859).

To answer the first research question comparing temporal trends in mean yield and yield variance across fertilizer × liming treatments, we fitted for each plot (‘fertilizer × liming’ treatment combination) a smoothing spline for the mean yield trend via a random-effects specification as a mixed model (Verbyla et al. 1999). An advantage of the smoothing spline approach over other regression approaches is that no specific assumption is needed as regards the functional form of the trend. Preliminary inspection of the data revealed that such flexibility was needed. The entire model syntax (using R version 4.0.0) is provided in Table A6 Supplementary material. The trends were fitted using ASReml-R using the specification random = ~spl(*t*,*k* = 10), where *t* is continuous time in years and *k* is the number of evenly spaced knots (set to 10, approximately 5-year intervals). For each of these 10 sub-periods, a separate residual variance was fitted to assess changes in temporal yield variance, with lower values indicating less unexplained variability between years (=more stable yields). Each ‘fertilizer × liming’ treatment combination was assumed to have a specific set of variance components ([t/ha]^2^) for the different periods. We denote the period-specific yield variances as ‘environmental variance’, a term coined by Römer (1917). Here, we replace the arithmetic treatment mean with the spline estimate of the temporal trend. The model, therefore, allowed yield variance to be determined independently of yield level, which highlights that the statistical approach used here differs from earlier approaches based on the PGE, which used the classic coefficient of variation for a measure for yield variability (Dodd et al. 1997). This is an important improvement because yield variance can be incorrectly interpreted if there is a systematic dependency of the measure of variation on the mean. No such dependencies were found in this analysis. The model was fitted separately for each ‘fertilizer × liming’ treatment. In this analysis, any systematic year effects (trend, etc.) are captured by the fitted splines, whereas the random residual captures any unexplained year effects.

To answer the second research question of how much of the variation in yield can be explained by climatic drivers and the interaction with agronomic management, treatment-specific multiple regression analyses were performed. The strength of the relationship between the response variable ‘yield’ and several explanatory environmental abiotic covariates (as described in former Chapter 2.2), as well as the importance of each of the predictors to the relationship, was assessed. We further tested for significant differences in regression coefficients of the main effects between treatments by using a general linear model approach, but no significant differences between treatments were found. Furthermore, a novel criss-cross regression approach with extended Eberhart–Russell regression analyses (Eberhart and Russell 1966) was used, in which the environmental mean was modeled using the previously as significant selected environmental abiotic covariates ((1) accumulated days of water stress from March to October; (2) mean air temperature from May to June; (3) mean air temperature from July to August based on the multiple regression analyses; shown in Table 1). This new method allowed us to assess the treatment-specific yield sensitivity (or responsiveness) to variability in these climatic conditions. The Eberhart–Russell model was further fitted using the criss-cross regression approach proposed for Finlay–Wilkinson regression by Digby (2009) and extended for the mixed model version by Nabugoomu et al. (1999). This iterative scheme allowed for (i) regression of the environmental index on covariates, (ii) heterogeneity of variance for the independent deviations from the regression, and (iii) serial correlation of the residuals. The intercept of Finlay–Wilkinson regressions provided information about the general yield level of a treatment compared to others. The slopes of regression lines can be interpreted as ‘yield sensitivity’ to climatic perturbation (slope > 1: higher sensitivity; slope < 1: less sensitivity/better resilience). Treatments with a slope of approximately 1 showed an average yield reaction across the treatments, similar to the average response indicated by the environmental mean (reference, black regression line with a slope of 1; Fig. 5a–d). We would like to stress that this criss-cross regression approach for the extended Finlay–Wilkinson regression was specifically developed for the PGE and, to the best of our knowledge, constitutes a novel method. Briefly, the Finlay–Wilkinson regression model can be written as ${\eta }_{ij}={\alpha }_{i}+{\beta }_{i}{w}_{j}$, where ${\eta }_{ij}$ is the expected performance of the *i*th treatment in the *j*th environment, ${\alpha }_{i}$ and ${\beta }_{i}$ are intercept and slope (‘yield sensitivity’) for the *i*th genotype, and ${w}_{j}$ is the environmental mean of the *j*th environment. The environmental mean, in turn, is modelled by a linear regression as ${w}_{j}={\theta }_{0}+{\theta }_{1}{x}_{1j}+{\theta }_{2}{x}_{2j}+....+{\theta }_{p}{x}_{pj}$, where *x*_*hj*_ is the value of the *h*th covariate in the *j*th environment and ${\theta }_{0},\dots , {\theta }_{p}$ are regression parameters. A detailed description of the model and its estimation is provided in Table A7 Supplementary material.

**Table 1:** Treatment-specific multiple regression analyses for the dependent variable ‘yield’ and selected climatic drivers. Analyses based on data (1965–2018) of total yield (sum of 1st + 2nd cut). Abbreviations: VIF = variance influence factor (measures the strength of the correlation between the independent variables in regression analysis); regression coefficient B = slope ‘b’ of regression line (how much Y changes for each change in X); standardized coefficient Beta = analogous to the interpretation of ‘b’, except that Beta expresses change in standard scores. Climatic drivers with significant main effects were selected based on a preceding general linear model analysis: accumulated number of water stress days from March to October (limited plant available soil water content with expected drought stress) and mean air temperature (=Temp.) in summer months (Fig. 2). No significant differences in regression coefficients B between treatments were found. Treatment explanations are provided in Table A3 Supplementary material (FYM/PM = farmyard/poultry manure; N* = sodium nitrate).

Treatment	Fertilization	Liming	General model statistic			First selected covariable				Second selected covariable			
			Durbin Watson test	Adjusted R Square	VIF Collinearity	Covariable	Regression coefficient B	Standardized coefficient Beta	*p* value	Covariable	Regression coefficient B	Standardized coefficient Beta	*p* value
3	Nil	pH 7	2.02	0.29	1.00	Water stress	-0.02	-0.55	0.00	none selected	n/a	n/a	n/a
		pH 6	1.68	0.23	1.00	Water stress	-0.02	-0.49	0.00	none selected	n/a	n/a	n/a
		pH 5	1.62	0.29	1.00	Water stress	-0.02	-0.54	0.00	none selected	n/a	n/a	n/a
		no chalk	1.58	0.25	1.00	Water stress	-0.02	-0.50	0.00	none selected	n/a	n/a	n/a
7/2	P K Na Mg	pH 7	1.97	0.49	1.36	Water stress	-0.03	-0.47	0.00	Temp.July-Aug.	-0.66	-0.35	0 00
		pH 6	2.05	0.37	1.36	Water stress	-0.02	-0.37	0.01	Temp.July-Aug.	-0.63	-0.35	0 01
		pH 5	1.76	0.34	1.01	Water stress	-0.04	-0.52	0.00	Temp.Mar.-Apr.	0.96	0.35	0 00
		no chalk	1.77	0.25	1.00	Water stress	-0.03	-0.52	0.00	none selected	n/a	n/a	n/a
6	N1 P K Na Mg	pH 7	2.05	0.48	1.29	Water stress	-0.02	-0.42	0.00	Temp.July-Aug.	-0.76	-0.40	0 00
		pH 6	2.06	0.40	1.29	Water stress	-0.02	-0.40	0.00	Temp.July-Aug.	-0.70	-0.37	0.01
		pH 5	n/a	n/a	n/a	n/a	n/a	n/a	n/a	n/a	n/a	n/a	n/a
		no chalk	n/a	n/a	n/a	n/a	n/a	n/a	n/a	n/a	n/a	n/a	n/a
9/2	N2 P K Na Mg	pH 7	1.71	0.36	1.36	Water stress	-0.02	-0.40	0.00	Temp.July-Aug.	-0.79	-0.31	0.02
		pH 6	1.51	0.36	1.36	Water stress	-0.02	-0.39	0.01	Temp.July-Aug.	-0.64	-0.38	0.01
		pH 5	1.70	0.35	1.36	Water stress	-0.02	-0.36	0.01	Temp.July-Aug.	-0.74	-0.35	0.01
		no chalk	1.57	0.38	1.36	Water stress	-0.02	-0.40	0.00	Temp.July-Aug.	-0.85	-0.32	0.02
11/1	N3 P K Na Mg	pH 7	1.64	0.29	136	Water stress	-0.02	-0.32	0.02	Temp.July-Aug.	-0.73	-0.33	0.02
		pH 6	1.62	0.27	136	Water stress	-0.02	-0.29	0.04	Temp.July-Aug.	-0.67	-0.34	0.02
		pH 5	1.71	0.26	136	Temp.July-Aug.	-1.30	-0.52	0.00	Waterstress	-0.02	-0.25	0.05
		no chalk	1.82	0.35	136	Temp.July-Aug.	-1.47	-0.60	0.00	Waterstress	-0.01	-0.16	0.05
13/2	FYM/PM	pH 7	1.72	0.34	136	Temp.July-Aug.	-0.91	-0.40	0.00	Waterstress	-0.02	-0.29	0.03
		pH 6	2.16	0.38	136	Temp.July-Aug.	-0.91	-0.44	0.00	Waterstress	-0.02	-0.29	0.03
		pH 5	1.72	0.33	136	Water stress	-0.03	-0.39	0.01	Temp.July-Aug.	-0.67	-0.30	0.03
		no chalk	1.64	0.21	1.00	Water stress	-0.03	-0.47	0.00	none selected	n/a	n/a	n/a
17	N*1	pH 7	1.95	0.31	1.19	Water stress	-0.02	-0A2	0.00	Temp.May-June	-0.25	-0.27	0.04
		pH 6	1.91	0.23	136	Water stress	-0.01	-0.29	0.04	Temp.May-June	-0.24	-0.29	0.05
		pH 5	1.80	0.32	136	Temp.May-June	-0.25	-0.58	0.00	Waterstress	-0.02	-0.34	0.01
		no chalk	1.73	0.28	1.00	Temp.July-Aug.	-0.58	-0.46	0.00	Waterstress	-0.01	-0.22	0.05

Regarding the third research question, we aimed to identify the potential correlation between plant species diversity (number of species and Shannon’s diversity index) and the mean yield or temporal yield variance depending on the agronomic treatment (fertilizer × liming). For this correlation analysis, we used the mean yield and temporal yield variance data based on the statistical analyses as described above to account for the experimental design and other covariates. With regard to the nonnormal distributions of the underlying data (outliers), correlation coefficients were calculated from the ranks of the data, not from their actual values. Kendall’s tau (τ) was used to ensure that the results were accurate because the same ranks were repeated too many times in the partial datasets (e.g. botanical surveys). The strength of the correlation increased both from 0 to +1 and 0 to −1, where −1/+1 indicated the strongest correlation and 0 indicated no correlation. The sign of *τ* showed the direction of the correlation; if negative, the variables were inversely related. To gain further insight into the temporal dynamics of the plant communities, a multivariate analysis was done using the data on relative biomass recorded on all study plots for the 10-year period (1991–2000) for which data were available. Although this only covered part of the total period covered by the yield variance analysis, it included a large range of yields. Two partial canonical correspondence analyses (pCCA) were done after removing species that were recorded in less than 5% of samples to avoid bias owing to rare species. First, the effect of year was analysed, including plot as a covariate. Second, the variance in community composition explained by plot was quantified, including year as a factorial covariate. In both cases, the proportion of functional group (grass/forb/legume) was included in the ordination plots as a supplementary variable.

## Results and discussion

### Temporal trends in yield variance and identifying main climatic drivers

Based on the unique long-term dataset and the new designed statistical approach, this study provides, for the first-time, insights into trends in temporal yield variance of grassland in response to climatic drivers and its dependence on agronomic management; yield variance was determined independently of yield level, which is important to avoid any misinterpretation of resilience. Our work adds a new perspective to earlier productivity analyses of grassland experiments, which were based mostly on shorter time periods (Prieto et al. 2015; Tilman et al. 2001; Graux et al. 2013; Trnka et al. 2021; Storkey et al. 2015; Haughey et al. 2018; Sanderson 2010), and reveals the importance of long-term experiments for detecting possible trends over time. It is complementary to a recent analysis of trends in productivity on the PGE that showed a consistent decline in yields in response to climate change across a longer time period (1902–2016) for four treatments and using yield data from just the first hay cut (Addy et al. 2022). By focusing on yield variance (using data from both cuts) across a wider range of treatments, our study provides additional insights into the potential for management to impact adaptability and resilience of grasslands to abiotic stress. Our treatment-specific model allowed temporal trends, using a spline component, to be dissected from residual variance, fitted as separate parameters for ten consecutive sub-periods.

**Fig. 3:**
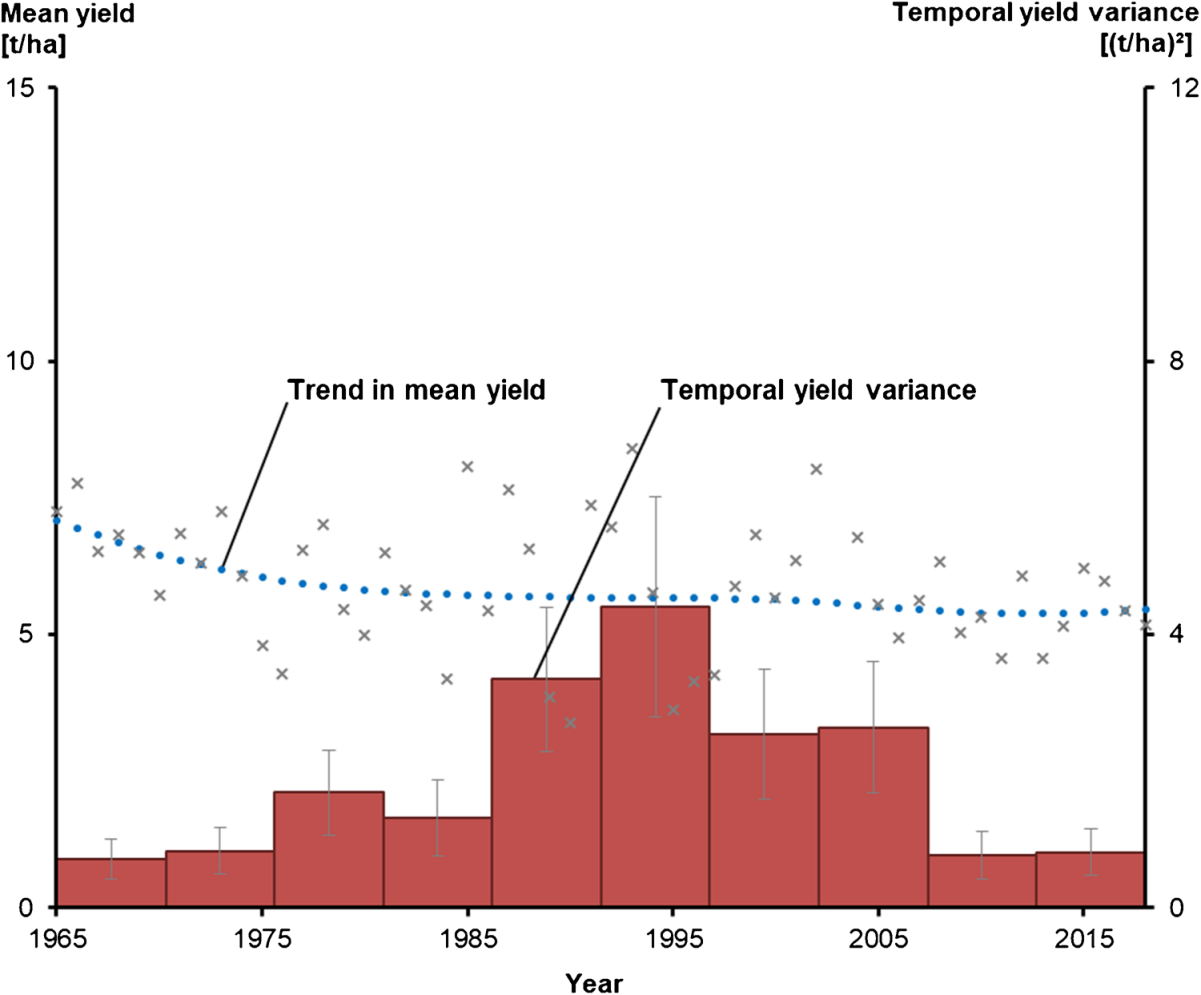
Summary plot for the overview temporal trend in mean yield (blue dotted line; grey crosses real harvest data) and yield variance (red bars) including standard errors (grey error bars) based on the total mean over all liming × fertilization treatments (1965–2018). Underlying plot specific results are provided in Fig. 4; overview of liming treatments (Fig. A8 Supplementary material), and overview of fertilization treatments (Fig. A9 Supplementary material). Detailed information about treatments is shown in Table A3 Supplementary material.

**Fig. 4:**
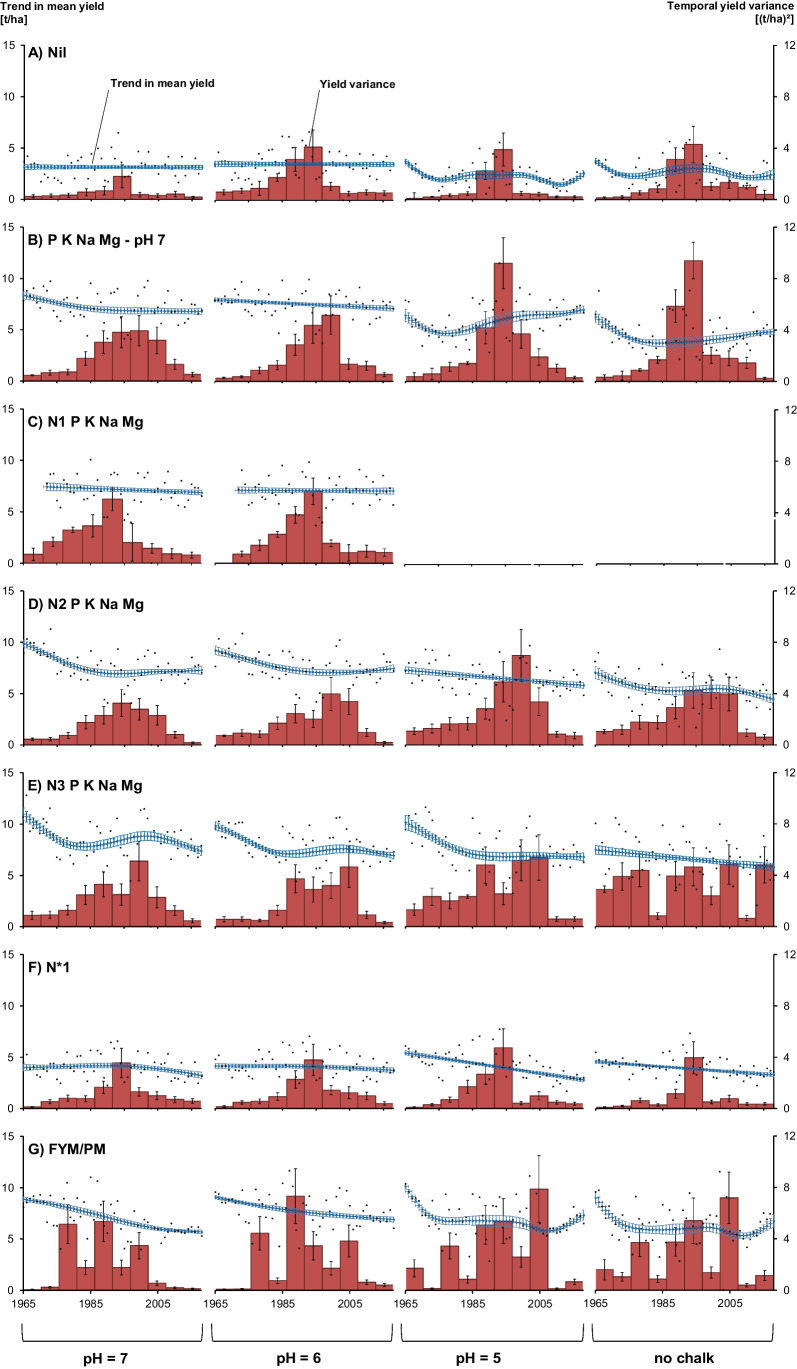
Temporal trends in mean yield (blue splines with approximated confidence intervals) and temporal yield variance (red bars incl. standard errors) depending on the treatment (fertilizer × liming) for the experimental period of 1965–2018. Analysis based on year × plot specific yields; these raw yield data are shown as black dots in each graphic. **A **Treatment no. 3: Nil (no fertilizer input)—pH 7/6/5/no chalk. **B** Treatment no. 7/2: P K Na Mg—pH 7/6/5/no chalk. **C** Treatment no. 6: N1 P K Na Mg—pH 7/6 (restricted data availability: only 1972–2018 and pH 7/6). **D** Treatment no. 9/2: N2 P K Na Mg—pH 7/6/5/no chalk. **E **Treatment no. 11/1: N3 P K Na Mg—pH 7/6/5/no chalk. **F** Treatment no. 17: N*1 (N* = sodium nitrate)—pH 7/6/5/no chalk. **G** Treatment no. 13/2: FYM/PM (farmyard/poultry manure)—pH 7/6/5/no chalk. Yield variance denoted Römer’s environmental variance, with lower values indicating more stable yields and higher values indicating more variable yields. Detailed information about treatments is shown in Table A3 Supplementary material. Summary plots are provided as an overall overview (Fig. 3), overview of liming treatments (Fig. A8 Supplementary material), and overview of fertilization treatments (Fig. A9 Supplementary material).

As a striking finding, we identified a similar pattern of temporal trends in yield variance (overview results Fig. 3) (Römer 1917), particularly for plots with low rates of N (48 kg N ha^−1^, N1) or no N fertilizer ‘Nil’ (plot specific results Fig. 4). This pattern can be described as follows: in the 1960s and 1970s, the plots showed relatively low yield variance. In subsequent years, we observed greater yield variance with a peak at approximately 1995 before becoming more stable in the past two decades (Fig. 3). This pattern of temporal trends in yield variance appeared to be less pronounced in treatments with a high soil pH; in contrast, peaks were more pronounced in treatments with low pH (Fig. A8 Supplementary material). The exceptions were treatments with reduced or no liming, but with a high mineral N supply (Fig. 4e) or farmyard manure (FYM) (Fig. 4g), where these patterns were not as evident, and periods of high yield variability were observed throughout the study period.

Key message for ‘Research question I’: There were consistent unimodal trends observed in yield variance in plots with low to moderate or no nitrogen fertilizer additions, with a peak in 1990s, after which variability declined. Yield was most variable in plots with higher nutrient inputs and lower soil pH.

Based on treatment-specific multiple regression analyses including environmental abiotic covariates, we explored the possible causes of the observed temporal trends in yield variance. We identified the accumulated days of water stress from March to October and the mean air temperature from July to August as the two main climatic drivers, explaining around one-third of the observed yield variance across treatments, or even up to 49%/48% of yield variance in treatment P K Na Mg and N1 P K Na Mg, respectively (Table 1 and Fig. 2). Overall, the impact of temperature driving yield variance was lower than the stronger impact of water stress (Table 1: see columns ‘standardized coefficient Beta’ with lower absolute values indicating less impact). The identified main climatic drivers tally with previous analyses of the PGE made by Dodd et al. (1994, 1997) and recent ones by Addy et al. (2021, 2022); any differences can be explained by the fact that we included the second cut in the yield data. The seasonality of temperate grassland production is primarily affected by soil moisture and temperature, which constrain the length and determine the intensity of the growing season (Trnka et al. 2011). Generally, significant temperature changes, particularly hot temperatures in summer with a limiting water balance, have a negative effect on grassland productivity (Kipling et al. 2016; Höglind et al. 2013) and are expected to increase the interannual and seasonal production variability of grassland systems (Graux et al. 2013; Chang et al. 2017). However, the water stress index we calculated is partly based on a derived meteorological (potential evaporation from grass) and an estimated parameter (soil water storage), which may differ from actual measurements. This may have contributed to the lower correlations seen in the Nil (no fertilizer) and N*1 plots together with the fact that other nutrients may have limited growth. Furthermore, water stress often occurs in July–August, so the fact that both factors come out as important may indicate that plants may be hit harder by high temperatures when transpiration and thereby cooling are limited. The effect of water stress was consistently present in all treatments and can be assumed to be the main driving factor in the PGE. Regarding the effect of agronomic practices on resistance to water stress (Table 1 and Fig. 2), sufficient liming is important for sustaining grassland productivity due to positive effects on soil pH, root growth, soil structure, nutrient availability, soil carbon, and soil biota (Holland et al. 2018; Fornara et al. 2011).

Key message for ‘Research question II—part A’: The accumulated days of water stress from March to October and mean air temperature from July to August were the most important climatic drivers, explaining approximately one-third of the observed interannual yield variance.

The remaining unexplained yield variance in the PGE might be driven by different environmental factors, which could not be determined further (i.e. due to lack of data in this study). A recent study by Addy et al. (2021) identified clusters of years with similar weather patterns between 1900 and 2020 at Rothamsted, which might help to clarify the unexplained rest of unimodal yield variance. They found a climate cluster characterized by cool and dry springs from approximately the 1960 to 1970s (in our study: stable yields were observed at the beginning), followed by a period with a variety of clusters and widely varying weather patterns (in our study: more variable yields occurred from approximately 1980 to 2000), and a transition since 2000 with an increased tendency toward higher temperatures in springs and drier periods in June (in our study: associated with more stable yields at the end; Fig. 3). The study suggests that the positive effects of sufficient water availability can offset the negative effects of warmer temperatures on pasture performance (Addy et al. 2021). An additional explanation could be that this peak in yield variance between 1980 and 2000 coincides with a period of decreasing nitrogen deposition and SO_2_ emissions (see Fig. A5 Supplementary material), which is reflected in shifts in plant community composition, especially an increase in the relative proportion of legumes. Regarding the SO_2_ emissions, there was a decline from approximately 65 kg ha^−1^ in 1980 to 5 kg ha^−1^ in 2006 (Anon 2006). We might expect this to affect species diversity only in S-limited plots, but S is applied together with K, Na, and Mg in most plots (such as in the ‘P K Na Mg’ treatment) in the PGE, so any effect of changes in air chemistry is more likely a result of decreasing N deposition in these plots. The highest peak in yield variance in the middle period was observed in plots lacking N but supplied with P, K, Na, and Mg (Fig. A9). These plots have the highest proportion of legumes (Table 2) that are sensitive to changes in N deposition. This suggests that where yield is dependent on biological N fixation (see key message IV), resilience will also be determined by the specific response of legumes to environment change—such as atmospheric N deposition (Storkey et al. 2015).

**Table 2:** Overview results for treatment-specific mean yield, temporal yield variance, and plant species diversity. n/a: no data available. Mean values based on available years of surveys (soil pH: 1998–2014; yield: 1965–2018; Shannon’s diversity index: 1991–2000, 2010–2012; species number: 1974, 1991–2000, 2010–2012; proportion legumes: 1991–2000). Treatment explanations are provided in Table A3 Supplementary material (FYM/PM = farmyard/poultry manure; N* = sodium nitrate).

Treatment	Fertilization	Liming	Soil pH	Mean yield [t/ha]		Yield variance [(t/ha)^2^]		Shannon's diversity index	Species number	Proportion legumes [%]
				Parameter estimate	Standard error	Parameter estimate error	Standard error			
3	Nil	pH 7	7.2	3.14	0.16	1.11	0.21	2.6	31	5.8
		pH 6	6.3	3.52	0.18	1.51	0.35	2.7	30	3.6
		pH 5	5.1	2.12	0.23	0.93	0.26	2.1	27	1.1
		no chalk	5.2	2.67	0.29	1.40	0.12	2.2	26	0.3
7/2	P K Na Mg	pH 7	7.0	7.02	0.26	2.10	0.17	2.4	23	28.6
		pH 6	6.2	7.46	0.16	1.97	0.15	2.3	23	16.5
		pH 5	5.2	5.94	0.35	2.59	0.19	2.3	22	25.7
		no chalk	5.0	4.15	0.32	2.63	0.16	2.1	23	17.4
6	N1 P K Na Mg	pH 7	7.0	7.16	0.24	1.94	0.41	2.5	22	17.1
		pH 6	5.9	7.06	0.24	2.01	0.42	2.0	20	11.3
		pH 5	n/a	n/a	n/a	n/a	n/a	n/a	n/a	n/a
		no chalk	n/a	n/a	n/a	n/a	n/a	n/a	n/a	n/a
9/2	N2 P K Na Mg	pH 7	7.1	7.32	0.26	1.62	0.21	2.1	17	2.7
		pH 6	6.2	7.37	0.26	1.82	0.19	1.8	17	1.6
		pH 5	5.0	6.45	0.22	2.69	0.22	1.5	15	10.8
		no chalk	3.6	5.39	0.35	2.38	0.29	0.6	3	0.0
11/1	N3 P K Na Mg	pH 7	7.0	8.45	0.38	2.18	0.31	1.5	11	0.0
		pH 6	6.1	7.54	0.30	1.99	0.19	1.6	12	0.0
		pH 5	5.1	7.16	0.36	2.75	0.27	0.8	10	0.0
		no chalk	3.5	6.54	0.27	3.38	0.41	0.0	2	0.0
13/2	FYM/PM	pH 7	6.9	7.07	0.21	1.87	0.18	2.3	20	0.6
		pH 6	6.0	7.75	0.19	2.27	0.23	2.3	20	4.6
		pH 5	5.3	6.86	0.35	2.79	0.39	2.3	20	0.2
		no chalk	5.1	6.17	0.40	2.85	0.27	2.0	19	0.5
17	N*1	pH 7	7.1	3.95	0.22	1.07	0.25	2.2	23	1.6
		pH 6	6.3	4.03	0.18	1.25	0.22	2.1	24	0.1
		pH 5	5.8	4.11	0.14	1.24	0.17	2.0	22	0.0
		no chalk	5.8	3.92	0.12	0.82	0.11	2.2	24	0.0

In addition to yield variance, the temporal trends in mean yield were in some cases relatively stable, as for the unfertilized ‘Nil’ treatment with a relatively constant yield of approximately 3 t ha^−1^ 286 (Fig. 4a). In other cases, the mean yield decreased over time (negative trends), supporting the conclusions of Addy et al. (2022), who analysed PGE data over a longer time period (1902–2016) and modelled spring hay yields under four fertilizer regimes in response to seasonal temperature and rainfall. The PGE modelling study showed that warmer and drier years in the twentieth and twenty-first centuries resulted in yield reductions and are forecasted to decline further up to 50% under future (2020–2080) climate scenarios (Addy et al. 2022).

This was the case particularly for treatments with greater inputs of N and those provided with organic manures, including the ‘N3 P K Na Mg—pH 7 or 6’ (Fig. 4e) and the ‘FYM/PM—pH 7 or 6’ (Fig. 4g) treatments in which mean yields decreased from >9 t ha^−1^ to nearly 5 t ha^−1^ from 1965 to 2018. In the PGE, the higher input plots (see ‘N3 P K Na Mg—pH 5 or no chalk’) were dominated by only a few grass species (Table 2), which might have resulted in greater yield variance when the climatic conditions were unfavourable for these species and reduced adaptability. Baca Cabrera et al. (2021) reported that the declining yields observed in grass-rich plant communities of the PGE over the last century were associated with decreases in N uptake, stomatal conductance, and transpiration, as affected by increasing temperature (Fig. A2 Supplementary material) and atmospheric CO_2_ concentrations (*C*_a_) (Fig. A4 Supplementary material). It was noted that grasses appeared to be more sensitive to increasing *C*_a_ than forbs, resulting in lower water use efficiency, decreased N uptake, and declining biomass production in grass-rich communities (Baca Cabrera et al. 2021). This indicated that plant communities comprising only a few species may be disproportionately affected if the species present are poorly adapted to changing climatic conditions and are also less adaptable to long-term climatic trends (Eisenhauer et al. 2019). Our results on trends in yield variance and mean yield support this hypothesis (Table 2 and Fig. 4).

### Determine yield sensitivity to climatic changes using a novel criss-cross regression analysis

**Fig. 5:**
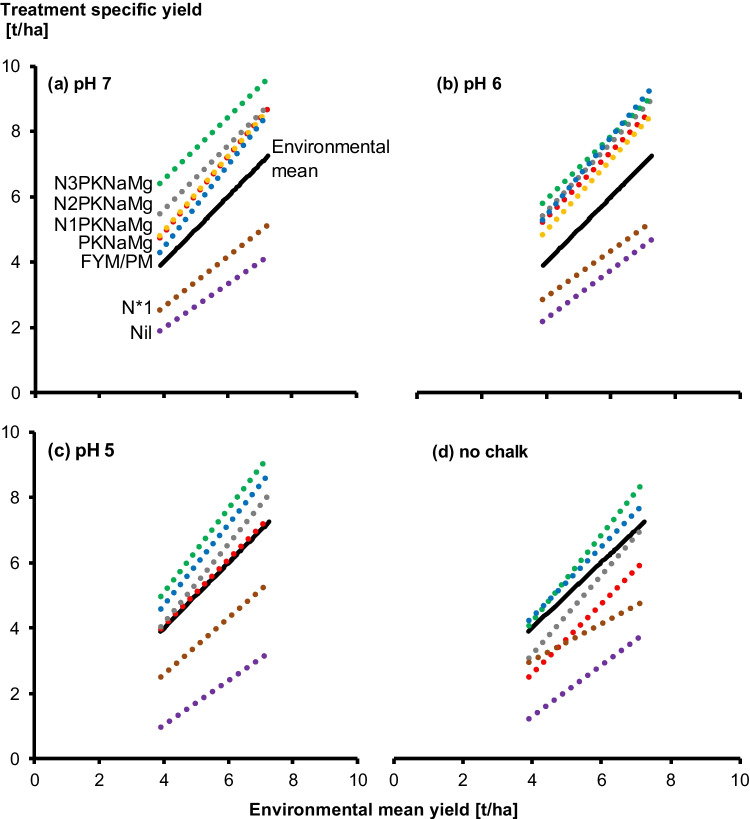
Graphical visualization of the treatment-specific criss-cross regression analyses (see Table 2; detailed explanation provided in Table A7 Supplementary material) depending on liming. **a** Treatments with pH 7. **b** Treatments with pH 6. **c** Treatments with pH 5. **d** Treatments with no chalk. The calculation of the environmental mean considered the three selected climatic drivers: the ‘accumulated number of water stress days’ and ‘mean air temperature from May–June and July–August’ (Fig. 2 and Table 1) were used to obtain predicted mean yields for each year (=environmental mean, *x*-axis) and regressed on the fertilization × liming treatment yields (*y*-axis); getting regression lines with treatment-specific intercepts and slopes. Treatment explanations are provided in Table A3 Supplementary material (FYM/PM = farmyard/poultry manure; N* = sodium nitrate).

We present a novel criss-cross regression analysis (extended Finlay–Wilkinson regression), in which we modeled the environmental mean using the three main identified climatic factors (Table 3 and Fig. 5). This approach allowed us to determine the treatment-specific yield sensitivity to variation in climatic conditions. A great advantage of this approach is its parsimony, providing a single slope for each treatment that assesses sensitivity to all included covariates simultaneously. The interaction between fitted environmental mean and treatments was highly significant (*F* = 3.17; *P* < 0.0001; Table A7 Supplementary material), showing that the slopes are different between treatments. The results shown in Table 3 can be interpreted as follows: a lower slope indicates less sensitivity (or responsiveness) of yield to variation in climatic conditions; a lower variance of deviation indicates a more stable yield. A higher slope indicates a higher sensitivity of yield to changing climatic conditions; a higher variance of deviation indicates more yield fluctuations. Regarding the predicted environmental mean, the signs of all three regression coefficients (theta1–theta3) were negative, meaning the predicted environmental mean increases with decreasing values for x1, x2, and x3. Thus, less water stress (x1) and lower temperatures from May to June (x2) and from July to August (x3) resulted in higher grassland yields during the observed experimental period.

**Table 3:** Treatment-specific criss-cross regression analyses (see Fig. 5; detailed explanation Table A7 Supplementary material). The calculation of the environmental mean considered the three selected climatic drivers (x1 ‘accumulated number of water stress days’; x2 ‘mean air temperature from May to June’; x3 ‘mean air temperature from July to August’ (Fig. 2 and Table 1), and were used to obtain predicted mean yields for each year (=environmental mean, *x*-axis) and regressed on the fertilization × liming treatment yields (*y*-axis); getting regression lines with treatment-specific intercepts and slopes. Treatment explanations are provided in Table A3 Supplementary material (FYM/PM = farmyard/poultry manure; N* = sodium nitrate).

Treatment	Fertilization	Liming	Criss-cross regression analyses				
			Slope		Intercept		Variance of deviation
			Parameter estimate	Standard error	Parameter estimate	Standard error	Parameter estimate
3	Nil	pH 7	0.70	0.14	-1.21	0.96	0.04
		pH 6	0.76	0.14	-1.18	0.95	0.03
		pH 5	0.69	0.15	-1.93	1.01	0.12
		no chalk	0.88	0.16	-2.74	1.06	0.20
7/2	P K Na Mg	pH 7	1.18	0.17	-0.21	1.11	0.29
		pH 6	1.99	0.17	1.14	1.13	0.33
		pH 5	1.17	0.18	-1.16	1.16	0.39
		no chalk	1.02	0.17	-1.60	1.14	0.36
6	N1 P K Na Mg	pH 7	1.18	0.17	-0.11	1.12	0.30
		pH 6	1.07	0.18	0.38	1.14	0.33
		pH 5	n/a	n/a	n/a	n/a	n/a
		no chalk	n/a	n/a	n/a	n/a	n/a
9/2	N2 P K Na Mg	pH 7	0.93	0.17	1.86	1.14	0.34
		pH 6	0.98	0.18	1.55	1.17	0.42
		pH 5	1.21	0.17	-1.17	1.11	0.29
		no chalk	1.19	0.19	-1.90	1.27	0.62
11/1	N3 P K Na Mg	pH 7	1.02	0.21	2.21	1.35	0.83
		pH 6	0.88	0.17	2.41	1.14	0.36
		pH 5	1.09	0.20	0.76	1.29	0.69
		no chalk	1.36	0.25	-1.79	1.60	1.50
13/2	FYM/PM	pH 7	1.11	0.21	-0.01	1.37	0.87
		pH 6	1.19	0.22	0.31	1.43	1.02
		pH 5	1.20	0.20	-0.33	1.32	0.76
		no chalk	1.09	0.21	-0.32	1.36	0.83
17	N*1	pH 7	0.86	0.15	-1.41	0.98	0.08
		pH 6	0.77	0.15	-0.67	0.97	0.05
		pH 5	0.87	0.17	-1.20	1.09	0.25
		no chalk	0.62	0.16	0.19	1.07	0.22
Predicited environmental mean	x1 (Water stress days)		-0.02	0.00	14.66	1.45	n/a
	x2 (Temperature May-June)		-0.18	0.13	14.66	1.45	n/a
	x3 (Temperature July-August)		-0.64	0.11	14.66	1.45	n/a

Key message for ‘Research question II—part B’: Liming to attain a soil pH of 6–7 and moderate N supply (e.g. treatment ‘N2 P K Na Mg—pH 6/7) were identified as the most promising agronomic practices for sustaining yield under varying climatic conditions and for reducing yield sensitivity to abiotic stresses.

In the intensively fertilized treatment without liming ‘N3 P K Na Mg—no chalk’, the regression lines had a slope >1 (*b* = 1.32), which indicates a high yield sensitivity to abiotic stress—where water is limiting or there is heat stress in the summer, proportionally more of the potential yield is lost (Table 3 and Fig. 5d). In comparison, for treatment ‘N2 P K Na Mg—pH 6/7’, lower slopes (*b* = 0.95/0.99) of regression lines were found, suggesting less yield sensitivity (or responsiveness) to climatic perturbation (Table 3 and Fig. 5a, b). This confirms findings of short-term grassland studies showing that higher/intensive nutrient inputs often increase yield but can destabilize productivity (Hautier et al. 2014, 2020; Zhang et al. 2016, 2019; Crawley et al. 2005). Moreover, the results showed that plots with greater fertilizer inputs were less resistant to climatic perturbation (higher yield variance), which is in agreement with the findings of Deguines et al. (2014), who reported that relative adaptability decreased with increasing land use intensity.

In addition to slopes, the intercept of regression lines is the second important criterion of Finlay–Wilkinson regression analysis. The intercept provides information about the overall yield level of a treatment (Table 3 and Fig. 5), with a higher intercept indicating higher yield performance (e.g. treatment ‘N3 P K Na Mg—pH 6/7’) and a smaller (or more negative) value referring to treatments with low yield level (e.g. unfertilized treatment ‘Nil—no chalk’). Overall, the combined evaluation of slope, intercept, and variance of deviation can point to treatments that show a favourable combination of resistance to climatic perturbation (slope < 1) together with a high and stable yield level (high intercept, low variance of deviation). In this study, liming (pH 7/6) and moderate N supply (e.g. treatment ‘N2 P K Na Mg—pH 6/7’) were identified as the most important agronomic practices for sustaining yield under changing climatic conditions (Table 3 and Fig. 5).

A striking finding was that the position and slope of regression lines, except ‘Nil’ (unfertilized) and ‘N*1’ (with 48 kg N ha^−1^ sodium nitrate), showed the least differentiation between treatments in the liming variant ‘pH 6’ (Fig. 5b), suggesting a similar environmental adaptability and yield performance. The largest yield susceptibility to lower pH values was observed in the ‘P K Na Mg’ treatment (possibly explained by the specific response for legumes, see above), and the least reactivity was shown by the ‘N3 P K Na Mg’ treatment, followed by the ‘N2 P K Na Mg’ and FYM/PM’ treatments (Table 3 and Fig. 5).

### Correlation between plant species diversity and yield variance

All correlations were assessed via the non-parametric Kendall’s tau, which provides a robust measure of association. In the PGE, an increased plant species diversity, expressed as Shannon’s diversity index, number of species, and proportion of legumes, was found to be associated with lower, but more stable, biomass yields and vice versa (Table 2 and Table A10 Supplementary material), which is in line with findings of recent grassland studies (Haughey et al. 2018; Sanderson 2010). For mean yield and temporal yield variance, the correlations (Kendall’s tau) with plant species diversity indices across treatments were significantly negative (*P* < 0.05): for yield variance and Shannon’s diversity index (*τ* = −0.30), species number (*τ* = −0.30), and proportion of legumes (*τ* = −0.20); for mean yield and Shannon’s diversity index (*τ* = −0.15), species number (*τ* = −0.50), and proportion of legumes (*τ* = −0.18) (Table A10 Supplementary material).

Key message for ‘Research question III—part A’: Higher plant species diversity was correlated not only with more stable grassland yields, but also with lower yield levels.

In addition, the pCCA of the effect of year on plant community composition including plot as a covariate (Fig. A12 Supplementary material) explained 16.4% of total variance (*P* < 0.001). Years tended to cluster in discrete periods characterized by the dominance of different species supporting the idea of environmental perturbation promoting species coexistence. For example, *Crepis capillaris* appears to have been favoured by the environmental conditions in 1991 and 1992 and *Leontodon autumnalis* in 1999 and 2000. The first axis discriminated between years dominated by grasses and those dominated by forbs. These results imply that the productivity of diverse plots with a more balanced community in terms of the ratio of grasses to forbs will be more resilient to variability in the environment. The pCCA of the effect of ‘fertilizer × liming treatment’ (sub-plot) on plant community composition including year as a covariate explained 64.2% of total variance (*P* < 0.001) and discriminated between unlimed plots with higher N-input (e.g. 9/2d: ‘N2 P K Na Mg—no chalk’; 11/1d: ‘N3 P K Na Mg—no chalk’) that are dominated by grasses and the unfertilized plots (3b/a: ‘Nil—pH 6/7’) that had a higher proportion of forbs (Fig. A13 Supplementary material).

Although our results are correlative and plant biodiversity data were only available for a sub-set of years, they are supported by evidence showing that greater plant species richness and phylogenetic diversity in managed grasslands may enhance their resilience to climate change via enhanced asynchrony in the performance of co-occurring species and result in more stable biomass production in response to disturbance (Isbell et al. 2017; Eisenhauer et al. 2019; Hautier et al. 2020). This assumes that a large species pool is likely, by chance, to possess one or two stress-tolerant species that are able to resist abiotic stress (e.g. drought) (Kahmen et al. 2005). Such stress-tolerant species could compensate for less tolerant species and thus help stabilize productivity in grasslands, and this could become more important with respect to climate change (Loreau and de Mazancourt 2013; Trnka et al. 2021; Haughey et al. 2018). In the PGE, treatments with high proportions of legumes (‘P K Na Mg’ and ‘N1 P K Na Mg’) had mean yields close to those in plots with higher rates of inorganic N fertilizer while also maintaining higher species richness. However, the yield variance in those plots was similar to that in plots with moderate fertilizer inputs and sufficient liming (treatment ‘N2 P K Na Mg – pH 7’; Table 2). These results could be explained by the diversity of fast vs slow functional traits (Storkey and Macdonald 2022; Reich and Cornelissen 2014). Grassland communities dominated by slow species were found to stabilize biomass productivity by increasing mean yield relative to temporal yield variance (Craven et al. 2018).

Key message for ‘Research question III–part B’: Yield variance increased, and plant species diversity decreased with greater fertilizer inputs and reduced liming. Treatments with high proportions of legumes had mean yields close to those in plots with higher rates of inorganic N fertilizer while also maintaining higher species richness.

The relationship between stable productivity (low yield variance) and plant species diversity strongly varies depending on agronomic management and soil conditions (Bullock et al. 2001; Hector et al. 1999; Tracy and Sanderson 2004; Crawley et al. 2005; Storkey et al. 2015). For example, species diversity decreased sharply with reduced soil pH, particularly under enhanced N supply in the form of ammonium sulfate (Crawley et al. 2005). Acidification due to long-term application of ammonium sulfate on the PGE has selected for a few grass species tolerant of low pH and has significantly reduced the proportion of legumes on plot 11/1 (see Table 2, ‘N3 PK Na Mg—no chalk’, pH 3.5). This acidic plot was probably subject to physiological stresses imposed by low pH (Dodd et al. 1994) and drought effects resulting from aluminum toxicity effects on root growth (Kohler et al. 2016), which may have magnified temporal yield variance. Hence, liming is a very important management factor for stabilizing yields and maintaining legumes in soils prone to acidification (Fornara et al. 2011; Storkey et al. 2015), which in this study has also been proven relevant for supporting stable grassland productivity over time (Table 1 and Fig. 4) and under varying climatic conditions, particularly limiting the water balance and higher temperatures from July to August (Table 3 and Fig. 5).

## Conclusion

Overall, our analysis led to the conclusion that liming, followed by moderate nutrient supply, promoted plant species diversity, yield stability, and environmental adaptability and enhanced the long-term sustainability of grassland production (in terms of stable productivity, biodiversity, and reduced synthetic fertilizer inputs). The three research questions can be answered as the following:

I. Yes, there were temporal trends in interannual yield variance over the study period and these varied in relation to fertilizer and lime applications. In particular, there were consistent unimodal trends observed in yield variance in plots with low to moderate or no nitrogen fertilizer additions, with a peak in the 1990s, after which variability declined. Yield was most variable in plots with higher nutrient inputs and lower soil pH.
II. The accumulated days of water stress from March to October and mean air temperature from July to August were identified as the most important climatic drivers, explaining approximately one-third of the observed interannual yield variance. Yes, yield sensitivity to these climatic drivers depended on agronomic management. Liming and moderate N supply reduced yield sensitivity to abiotic stresses.
III. Yes, there was a correlation between plant species diversity, mean yield, and yield variance. Higher plant species diversity was correlated not only with more stable grassland yields but also with lower yield levels. Yield variance increased, and plant species diversity decreased with greater fertilizer inputs and reduced liming.

As a limitation of this study, it should be noted that there is a lack of replication in the PGE and that plot errors and ‘fertilizer × liming’ interactions (=residual) could not be separated. Furthermore, biodiversity data were only available for a sub-set of years and plots. For this reason, our findings should be interpreted carefully and validated by further detailed analyses including prospective yield data, biodiversity surveys, and soil–climate measurements.

As possible features of future studies, meta-analyses of various grassland LTEs under different climate and site conditions may provide further valuable information about temporal trends in yield variance depending on agronomic management. In addition to retrospective analyses, grassland LTEs are also a valuable source for application of agroecosystem models to simulate grassland responses under contrasting soil conditions and under future climate scenarios (Qi et al. 2018), which should be addressed more in upcoming studies.

Overall, the analysis of long-term grassland experiments, like this study based on the PGE, could help to improve the climate resilience and sustainability of grassland systems by identifying climatic drivers and optimizing the agronomic management accordingly. In particular, the application of the new designed criss-cross regression approach, in which the environmental mean was modeled using the selected environmental abiotic covariates, allows the assessment of the yield sensitivity (or responsiveness) to changes in climatic conditions. The application of this criss-cross regression approach in other agro-ecological trials could help to identify climatic drivers of production risk and to derive agronomic management strategies for improving the climate resilience of cropping systems. This will become increasingly important for stable agricultural production in the face of climate change and the associated growing risk for abiotic stresses.

## Supplementary Information

Below is the link to the electronic supplementary material.

Supplementary file1 (DOCX 324 KB)

Supplementary file2 (DOCX 158 KB)

Supplementary file3 (DOCX 21 KB)

Supplementary file4 (DOCX 35 KB)

Supplementary file5 (DOCX 115 KB)

Supplementary file6 (DOCX 21 KB)

Supplementary file7 (DOCX 68 KB)

Supplementary file8 (DOCX 61 KB)

Supplementary file9 (DOCX 73 KB)

Supplementary file10 (DOCX 20 KB)

Supplementary file11 (DOCX 152 KB)

## Data Availability

The yield dataset of the PGE and the mean annual air temperatures from 1965 to 2018 at Rothamsted were provided by the electronic Rothamsted Archive and are available from the e-RA website (see https://doi.org/10.23637/rpg5-yields1965-2018-01 and https://doi.org/10.23637/rms-RMAAtemp-02, respectively). The precipitation chemistry data from 1992 to 2015 were provided by the UK Environmental Change Network (ECN) and are publicly available (see https://doi.org/10.5285/18b7c387-037d-4949-98bc-e8db5ef4264c). The annual emissions of sulfur dioxide were provided by the UK National Atmospheric Emissions Inventory and are publicly available (https://naei.beis.gov.uk/data/data-selector?view=airpollutants). The species of grasses, forbs, and legumes comprising at least 5% of the aboveground biomass found in the surveys of the PGE are available from the e-RA website (see https://doi.org/10.23637/rpg5-species_1991-2000-01 and http://www.era.rothamsted.ac.uk/dataset/rpg5/01-OAPGspecies).

## Code availability

The R-scripts for the statistical analyses generated during the current study are available in Tables A6–A7 Supplementary material or from the corresponding author on reasonable request.
