# Supplementary material for: Long-term trends in yield variance of temperate managed grassland
Source: Agron Sustain Dev. 2023 Apr 26;43(3):37. doi: 10.1007/s13593-023-00885-w (PMC10133363; doi:10.1007/s13593-023-00885-w)
Supplement: Supplementary file 1 — Supplementary file1 (DOCX 324 KB) [file 13593_2023_885_MOESM1_ESM.docx]

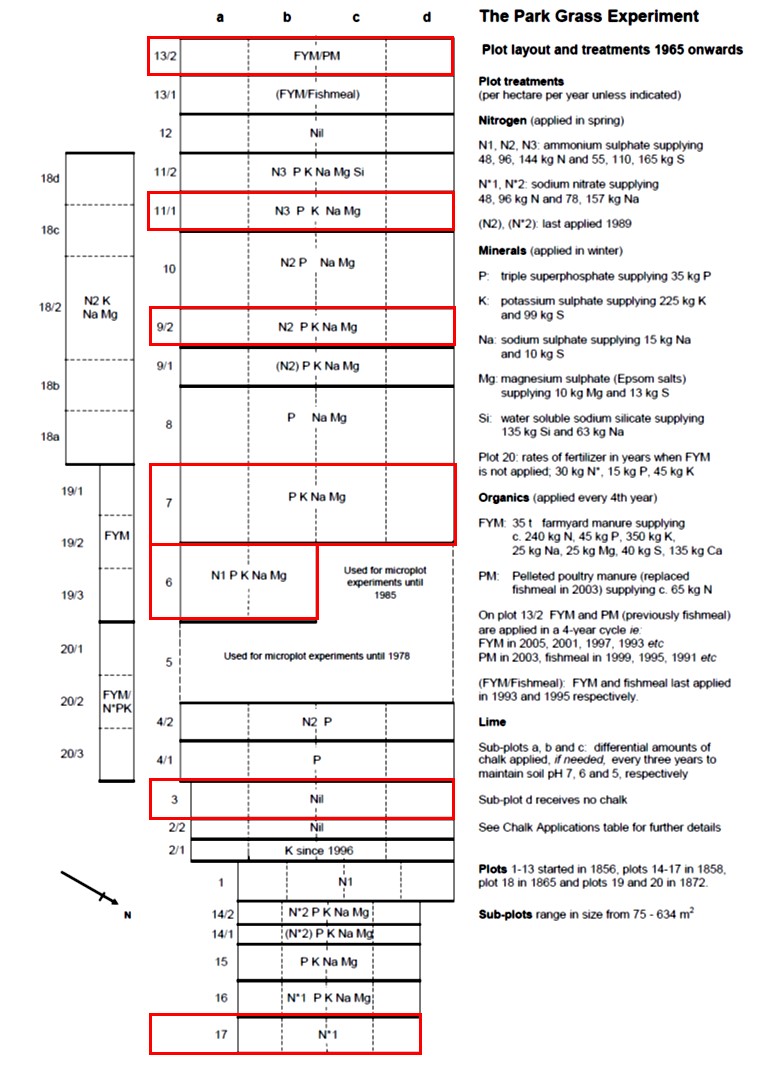

**Fig. A1-a Supplementary material** Plot layout and treatments of the Park Grass Experiment from 1965 onward. Red framed treatments were included in this study (treatment details provided in Table A3 Supplementary material). The liming treatments “a, b and c” received different amounts of chalk (CaCO_3_) when necessary to achieve and/or maintain the soil (0-23 cm) at pH 7, 6 and 5, respectively. Subplot “d” received no lime, and its pH reflects inputs from the various treatments and the atmosphere. Soils on the unlimed subplots “d” of the Nil treatments are now at approximately pH 5, while soils receiving 144 kg N ha^-1^ as ammonium sulfate are at approximately pH 4. Source: Rothamsted Research (2021).


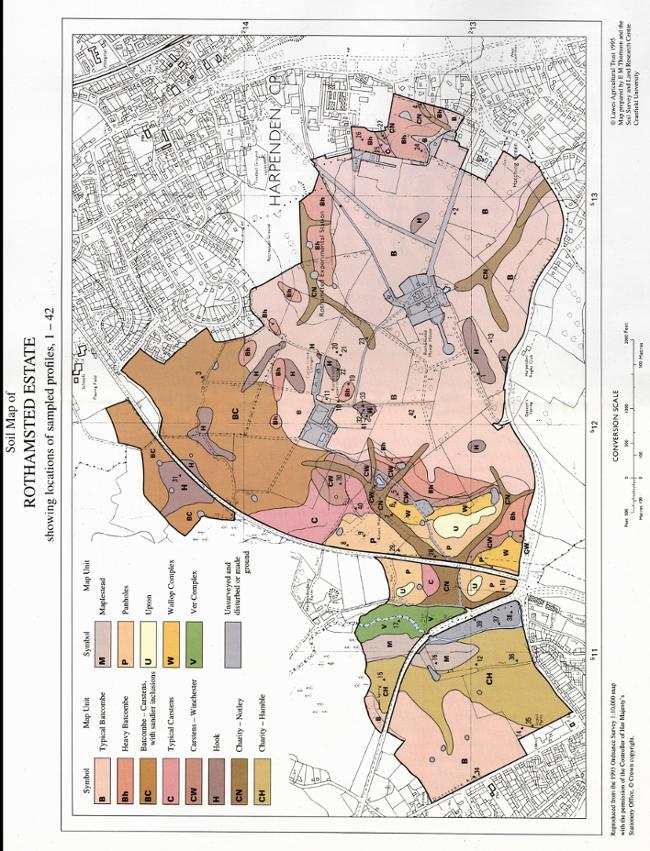


**Fig. A1-b Supplementary material** Soils at Rothamsted Colour map. The Park Grass Experiment is red framed. Source: Avery and Catt (1995) - online available: <https://doi.org/10.23637/ERADOC-1-143> (page 43).
