# Supplementary material for: Long-term trends in yield variance of temperate managed grassland
Source: Agron Sustain Dev. 2023 Apr 26;43(3):37. doi: 10.1007/s13593-023-00885-w (PMC10133363; doi:10.1007/s13593-023-00885-w)
Supplement: Supplementary file 2 — Supplementary file2 (DOCX 158 KB) [file 13593_2023_885_MOESM2_ESM.docx]

**
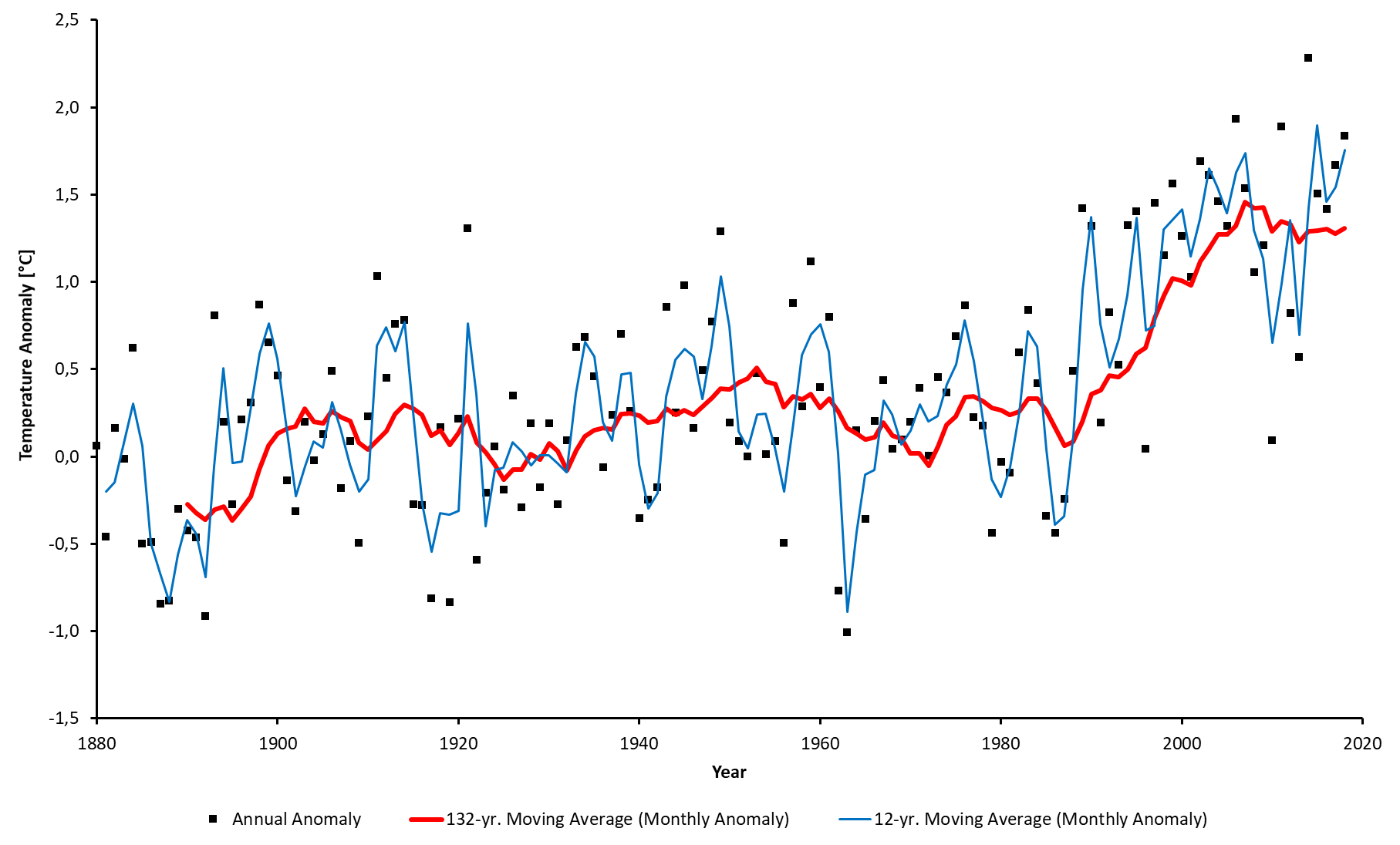

Fig. A2 Supplementary material** Temperature anomalies (1880-2018) shown as differences in the mean air temperature [°C] from the long-term mean for 1878 to 1920 (8.91 °C) at Rothamsted. Source: see doi 10.23637/rms-RMAAtempanomaly-1.
