# Supplementary material for: Long-term trends in yield variance of temperate managed grassland
Source: Agron Sustain Dev. 2023 Apr 26;43(3):37. doi: 10.1007/s13593-023-00885-w (PMC10133363; doi:10.1007/s13593-023-00885-w)
Supplement: Supplementary file 3 — Supplementary file3 (DOCX 21 KB) [file 13593_2023_885_MOESM3_ESM.docx]

| **No.** | **Treatment** | **Nutrient supply (units: per hectare per year unless indicated)** |
| --- | --- | --- |
| 3 | Nil | No fertilizer input; nutrient depletion |
| 7/2 | P K Na Mg | Triple superphosphate: 35 kg P  Potassium sulfate: 225 kg K + 99 kg S  Sodium sulfate: 15 kg Na +10 kg S  Magnesium sulfate: 10 kg Mg + 13 kg S |
| 6 | N1 P K Na Mg | Ammonium sulfate: 48 kg N + 55 kg S  Triple superphosphate: 35 kg P  Potassium sulfate: 225 kg K + 99 kg S  Sodium sulfate: 15 kg Na +10 kg S  Magnesium sulfate: 10 kg Mg + 13 kg S |
| 9/2 | N2 P K Na Mg | Ammonium sulfate: 96 kg N + 110 kg S  Triple superphosphate: 35 kg P  Potassium sulfate: 225 kg K + 99 kg S  Sodium sulfate: 15 kg Na +10 kg S  Magnesium sulfate: 10 kg Mg + 13 kg S |
| 11/1 | N3 P K Na Mg | Ammonium sulfate: 144 kg N + 165 kg S  Triple superphosphate: 35 kg P  Potassium sulfate: 225 kg K + 99 kg S  Sodium sulfate: 15 kg Na +10 kg S  Magnesium sulfate: 10 kg Mg + 13 kg S |
| 13_2 | FYM/PM | Farmyard manure 35 t applied every 4^th^ year: c. 240 kg N + 45 kg P + 350 kg K + 25 kg Na + 25 kg Mg + 40 kg S + 135 kg Ca  Pelleted poultry manure: 65 kg N |
| 17 | N*1 | Sodium nitrate: 48 kg N + 157 kg Na |

**Table A3 Supplementary material** Fertilizer treatments in the Park Grass Experiment included in this study (1965–2018). Source: Rothamsted Research (2021). Nitrogen applied in spring; Minerals applied in winter. See the plot layout and treatments in Fig. A1 Supplementary material. Note: Plot 7 was divided into 7/1 and 7/2 in 2013, and P was withheld on plot 7/1.
