# Supplementary material for: Long-term trends in yield variance of temperate managed grassland
Source: Agron Sustain Dev. 2023 Apr 26;43(3):37. doi: 10.1007/s13593-023-00885-w (PMC10133363; doi:10.1007/s13593-023-00885-w)
Supplement: Supplementary file 4 — Supplementary file4 (DOCX 35 KB) [file 13593_2023_885_MOESM4_ESM.docx]

**
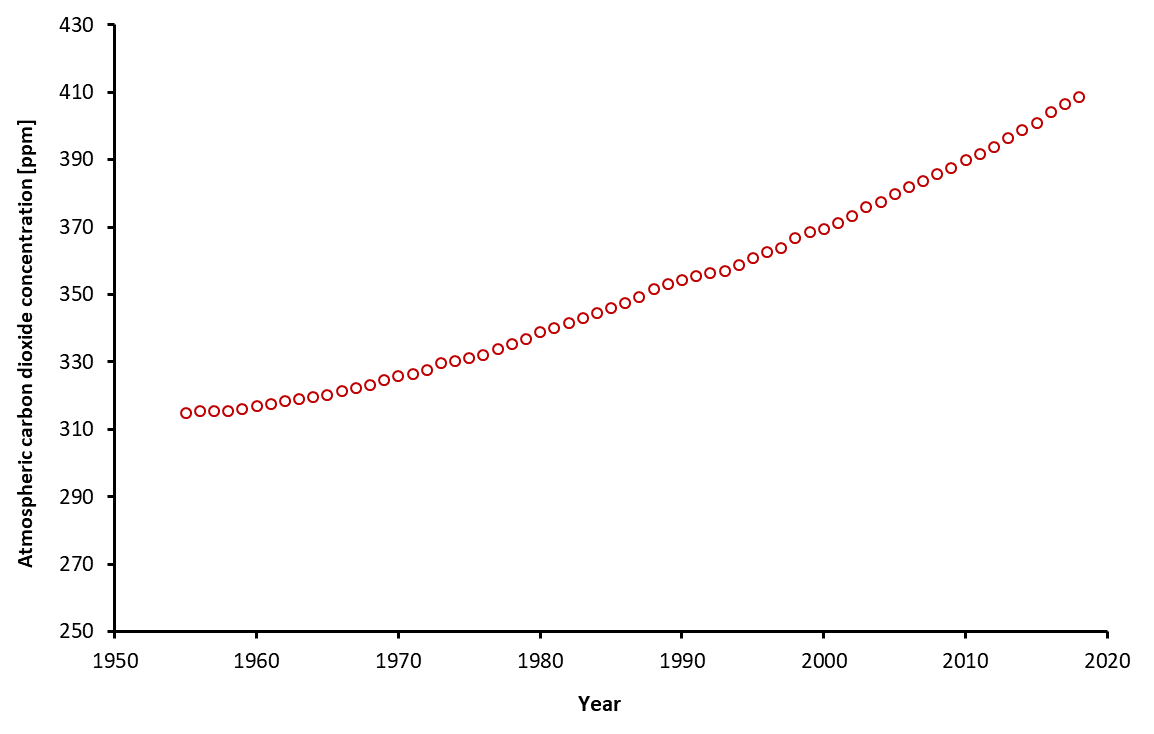
Fig. A4 Supplementary material** Temporal development of the mean global atmospheric carbon dioxide concentrations [ppm] (1965-2018). Measurements were collected by NOAA's Global Greenhouse Gas Reference Network (https://gml.noaa.gov/ccgg/about.html). Credit: NOAA Global Monitoring Laboratory.
