# Supplementary material for: Long-term trends in yield variance of temperate managed grassland
Source: Agron Sustain Dev. 2023 Apr 26;43(3):37. doi: 10.1007/s13593-023-00885-w (PMC10133363; doi:10.1007/s13593-023-00885-w)
Supplement: Supplementary file 5 — Supplementary file5 (DOCX 115 KB) [file 13593_2023_885_MOESM5_ESM.docx]

**
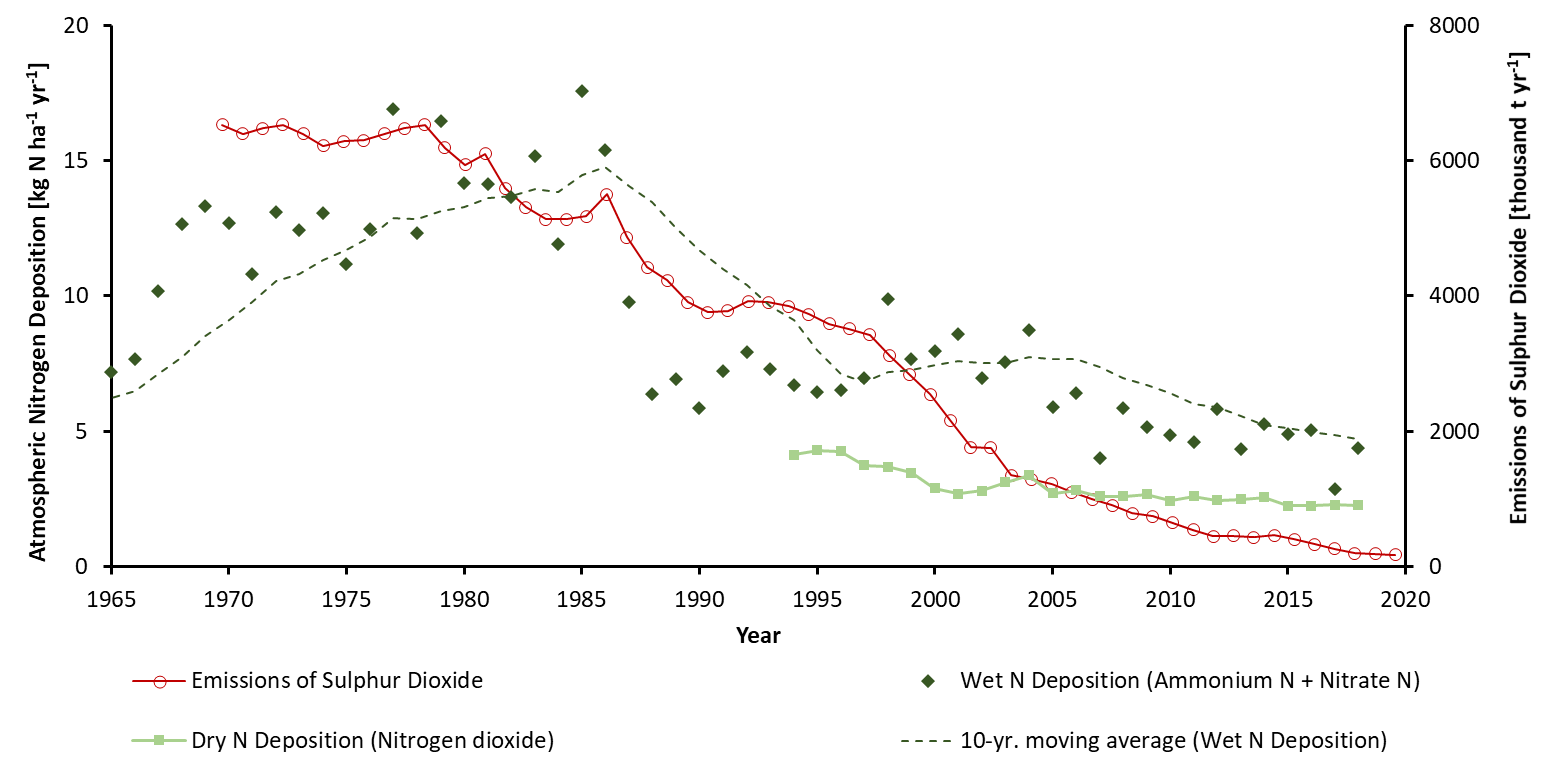

Fig. A5 Supplementary material** Temporal development of atmospheric nitrogen deposition at Rothamsted [kg N ha^-1^ yr^-1^] and emissions of sulfur dioxide in the UK [thousand t yr^-1^] (1965/1970-2018). UK-Inventory of sulfur dioxide emissions started 1970. Source for atmospheric nitrogen deposition: see doi 10.5285/18b7c387-037d-4949-98bc-e8db5ef4264c (UK Environmental Change Network). Source for emissions of sulfur dioxide: https://naei.beis.gov.uk/data/data-selector?view=air-pollutants (UK National Atmospheric Emissions Inventory).
