# Supplementary material for: Long-term trends in yield variance of temperate managed grassland
Source: Agron Sustain Dev. 2023 Apr 26;43(3):37. doi: 10.1007/s13593-023-00885-w (PMC10133363; doi:10.1007/s13593-023-00885-w)
Supplement: Supplementary file 6 — Supplementary file6 (DOCX 21 KB) [file 13593_2023_885_MOESM6_ESM.docx]

**Table A6 Supplementary material** Model syntax (R version 4.0.0) used for the statistical analysis of trends in mean yield and temporal yield variance (Park Grass Experiment, 1965–2018).

**__________________________________________________________________________________**

**require**(asreml)

data=readxl::read_xlsx("C:/Users/Hadasch/Downloads/Datensatz_Park Grass Experiment_ Rothamsted.xlsx", col_types = c("text","text","text","text","numeri c","numeric","numeric"))

data$fert_treat=as.factor(data$`Fertilizer_treatment (main plot)`)
data$fert_treat1=as.factor(data$`Fertilizer_treatment name (main plot)`)
data$limi_treat=as.factor(data$`Liming_treatment (sub plot)`)
data$fert_limi_treat=as.factor(paste(data$fert_treat,data$limi_treat,sep ="_"))
data$Year=as.factor(data$Year)
data$t=as.numeric(as.character(data$Year))
data$t_cent=(data$t-min(data$t))/max(data$t)
data$t_cent_sqrt=sqrt(data$t_cent)
data$y=as.numeric(data$`Total biomasss (1st+2nd cut)`)
print(length(unique(data$fert_limi_treat)))

data$period=NA
spl_knots=round(splinek(data$t, k = 10)$knotpoints)

## Spline: design points closer than 0.0053 have been merged.

spl_knots[1]=spl_knots[1]-1
**for**(k **in** 1:(length(spl_knots)-1))
which(is.na(data$period))

## integer(0)
fert_limi_treat=unique(data$fert_limi_treat)
fert_limi_treat=fert_limi_treat[order(fert_limi_treat)]

means=c()
vcs=c()
**for**(i **in** fert_limi_treat) {

 #i=fert_limi_treat[1]
 data_i=data[which(data$fert_limi_treat==i),]
 data_i$period=as.factor(as.character(data_i$period))
 asrSP <- asreml(y ~ t,
 random = ~spl(t,k=10),
 residual = ~dsum(~units|period),
 options=asreml.options(step.size=.316,maxit=50,workspace ="240mb",pworkspace="240mb"),
 data = data_i)

asrSP=update(asrSP) asrSP=update(asrSP) asrSP=update(asrSP) asrSP=update(asrSP) #asrSP=update(asrSP) #asrSP=update(asrSP)
 vcSP=summary(asrSP)$varcomp

 pSP=predict(asrSP,classify = "t",design.points = list(t = unique(data$t)))
 means_i=pSP$pvals#[which(preds$pvals$fert_limi_treat==i),]
 means_i$upper=(means_i$predicted.value+2*means_i$std.error)
 means_i$lower=(means_i$predicted.value-2*means_i$std.error)
 means=rbind(means,cbind(i,means_i))
 vcs=rbind(vcs,cbind(i,vcSP))
}
