# Supplementary material for: Long-term trends in yield variance of temperate managed grassland
Source: Agron Sustain Dev. 2023 Apr 26;43(3):37. doi: 10.1007/s13593-023-00885-w (PMC10133363; doi:10.1007/s13593-023-00885-w)
Supplement: Supplementary file 7 — Supplementary file7 (DOCX 68 KB) [file 13593_2023_885_MOESM7_ESM.docx]

**Table A7 Supplementary material** Criss-cross regression analyses (based on Finlay-Wilkinson regression approach) extended for environmental abiotic covariates (Park Grass Experiment, 1965–2018).

**__________________________________________________________________________________**

***The original model***

The Finlay-Wilkinson model assumes that the response of different treatments (genotypes in the original paper) in varying environments (years in our case) can be modeled using the linear predictor

$\eta_{ij}=\alpha_{i}+\beta_{i}w_{j}$ (1)

where $\eta_{ij}$ is the expected performance of the *i*-th treatment in the *j*-th environment (year), $\alpha_{i}$ and $\beta_{i}$ are intercept and slope for the *i*-th genotype and $w_{j}$ is the environmental mean of the *j*-th environment. Finlay and Wilkinson (1963) estimated $w_{j}$ by the arithmetic mean of all observed genotype mean yields, $y_{ij}$, i.e., they used . This does not yield the least-squares fit of (1), however. Digby (1979) showed how to obtain the least squares fit by alternating least squares, and Ng and Grunwald (1997; also see Ng and Williams, 2001) showed how to do this using nonlinear least squares. The model is not linear in the parameters, and some restriction on the parameters is needed for the multiplicative term $\beta_{i}w_{j}$, as will be detailed later.

***Modeling the environmental mean using environmental abiotic covariates***

A downside of Model (1) is that it cannot be used to predict the performance in unseen environments. If $w_{j}$ can be replaced by an observable covariate, such predictions become possible. However, a single covariate rarely provides good predictions. Thus, a natural extension is to perform a multiple regression on several covariates (Denis, 1988). Such a factorial regression model quickly becomes very complex because each treatment needs to have a separate regression coefficient for each environmental abiotic covariate. For these reasons, it is desirable to consider more parsimonious alternatives. Specifically, one may consider regressing $w_{j}$ on multiple covariates, i.e.,

$w_{j}=\theta_{0}+\theta_{1}x_{1j}+\theta_{2}x_{2j}+....+\theta_{p}x_{pj}$ (2)

where *x_hj_* is the value of the *h*-th covariate in the *j*-th environment and are regression parameters (Li et al., 2018; Guo et al., 2021). Inserting this into (1), we find

$\eta_{ij}=\alpha_{i}+\beta_{i}\left(\theta_{0}+\theta_{1}x_{1j}+\theta_{2}x_{2j}+....+\theta_{p}x_{pj}\right)$ (3)

The regression model is also not linear in the parameters, and there is an overparameterization that needs to be resolved. Several methods of estimation can fit Model (3). Here, we will first consider a method that is readily extended to allow for additional random effects, serial correlation and heterogeneity of variance, all of which are needed for the Park Grass data. The method we use is based on Digby (1979), who suggested an alternating least squares approach that iterates between two linear regressions, one for treatments, fixing year parameters, and the other one for years, fixing the treatment parameters. We may also refer to this approach as criss-cross regression, a term coined by Gabriel and Zamir (1979). Thus, in the crisis step, we may fix the treatment intercept and slopes in (3) and fit the year-mean regression parameters $\theta_{0},\theta_{1},\theta_{2},...,\theta_{p}$. In the cross-step, we may fix the year-mean regression parameters in (3) and estimate the *n* treatment-specific intercepts and slopes $\alpha_{i},\beta_{i} \left(i=1,...,n\right)$. In the case of balanced data, the scheme may be simplified as follows. Consider the environmental averages based on (3):

(4)

Thus, multiple regression of environmental means $\bar{\eta}_{\bullet j}$ on the covariates provides estimates of slopes for covariates $x_{kj}\left(k=1,...,p\right)$ and the intercept . Without loss of generality, we may then use

(5)

as our predictor for the environmental index in (1). This approach requires complete treatment-environment tables. Our data are nearly balanced; hence, we use this method as an approximation to explore the importance of different covariates. For example, we can run a classical multiple regression analysis with year means as the response to identify important climatic drivers. The following three climatic drivers were selected for the PGE: x1. accumulated days of water stress from March-October; x2. mean air temperature from May-June; x3. mean air temperature from July-August.

***Mixed-model extension of the model***

Following Nabugoomu et al. (1999), the criss-cross regression approach of Digby (1979) is easily extended in a mixed model framework. Here, we are interested in three aspects: (i) Model (3) represents only the systematic part of the response. The observed data will display heterogeneity of variance between treatments in the deviations from the regression line. This treatment-specific variance has been proposed by Eberhart and Russell (1966) as an additional stability parameter to the regression coefficient $\beta_{i}$. (ii) Plot errors on the same plot are expected to display serial correlation between years. (iii) A covariance is expected between treatments in the same year due to the shared environment. This can be modeled by a random year main effect. That effect is also expected to be serially correlated. Thus, our model for the response $y_{ij}$ of the *i*-th treatment in the *j*-th year is

$y_{ij}=\eta_{ij}+u_{j}+d_{ij}+e_{ij}$ (6)

where $\eta_{ij}$ is as defined in (3), $u_{j}$ is the random year main effect with variance $\sigma_{u}^{2}$ and correlation between years *j* and *j'*, $e_{ij}$ is the random plot error with variance $\sigma_{e}^{2}$ and correlation between years *j* and *j'*, and $d_{ij}$ is the independent random deviation from the regression with variance $\sigma_{d\left(i\right)}^{2}$ for the *i*-th treatment. When fitting this mixed model using criss-cross regression, we fix the variance parameters at their current estimates in the criss step because this only has *p*+1 parameters. These parameters are re-estimated in the cross-step using residual maximum likelihood.

**SAS output of the criss-cross analyses:**

The algorithm took 7 criss-cross iterations.

Solution for Fixed Effects

Effect Estimate Error DF t Value Pr > |t|

trt_slope 14.6601 1.4462 1360 10.14 <.0001

trt_slope*x1 -0.01858 0.003517 1360 -5.28 <.0001

trt_slope*x2 -0.1794 0.1266 1360 -1.42 0.1568

trt_slope*x3 -0.6381 0.1135 1360 -5.62 <.0001

Covariance Parameter Estimates

Cov Parm Subject Group Estimate

Year 0.6275

Year Treatment 11a_1st 0.8278

Year Treatment 11b_1st 0.3565

Year Treatment 11c_1st 0.6869

Year Treatment 11d_1st 1.4980

Year Treatment 13a_1st 0.8728

Year Treatment 13b_1st 1.0178

Year Treatment 13c_1st 0.7578

Year Treatment 13d_1st 0.8346

Year Treatment 17a_1st 0.07831

Year Treatment 17b_1st 0.05012

Year Treatment 17c_1st 0.2518

Year Treatment 17d_1st 0.2151

Year Treatment 3a_1st 0.04422

Year Treatment 3b_1st 0.03067

Year Treatment 3c_1st 0.1237

Year Treatment 3d_1st 0.2017

Year Treatment 6a_1st 0.2962

Year Treatment 6b_1st 0.3336

Year Treatment 7a_1st 0.2858

Year Treatment 7b_1st 0.3278

Year Treatment 7c_1st 0.3904

Year Treatment 7d_1st 0.3583

Year Treatment 9a_1st 0.3434

Year Treatment 9b_1st 0.4231

Year Treatment 9c_1st 0.2898

Year Treatment 9d_1st 0.6187

Variance Treatment 0.4878

AR(1) Treatment 0.8532

Fit Statistics

-2 Res Log Likelihood 3337.81787

AIC (Smaller is Better) 3395.81787

AICC (Smaller is Better) 3397.17513

BIC (Smaller is Better) 3452.95634

CAIC (Smaller is Better) 3481.95634

HQIC (Smaller is Better) 3417.79057

Solution for Fixed Effects

Standard

Effect Treatment Estimate Error DF t Value Pr > |t|

Treatment 11a_1st 2.2077 1.3531 1312 1.63 0.1030

Treatment 11b_1st 2.4102 1.1421 1312 2.11 0.0350

Treatment 11c_1st 0.7603 1.2946 1312 0.59 0.5571

Treatment 11d_1st -1.7850 1.5979 1312 -1.12 0.2641

Treatment 13a_1st -0.01498 1.3712 1312 -0.01 0.9913

Treatment 13b_1st 0.3070 1.4275 1312 0.22 0.8298

Treatment 13c_1st -0.3294 1.3244 1312 -0.25 0.8036

Treatment 13d_1st -0.3200 1.3559 1312 -0.24 0.8135

Treatment 17a_1st -1.4139 0.9849 1312 -1.44 0.1514

Treatment 17b_1st -0.6683 0.9657 1312 -0.69 0.4891

Treatment 17c_1st -1.2001 1.0874 1312 -1.10 0.2700

Treatment 17d_1st 0.1930 1.0672 1312 0.18 0.8565

Treatment 3a_1st -1.2146 0.9616 1312 -1.26 0.2068

Treatment 3b_1st -1.1769 0.9517 1312 -1.24 0.2165

Treatment 3c_1st -1.9254 1.0138 1312 -1.90 0.0578

Treatment 3d_1st -2.7368 1.0597 1312 -2.58 0.0099

Treatment 6a_1st -0.1141 1.1229 1312 -0.10 0.9191

Treatment 6b_1st 0.3759 1.1432 1312 0.33 0.7423

Treatment 7a_1st -0.2121 1.1056 1312 -0.19 0.8479

Treatment 7b_1st 1.1396 1.1275 1312 1.01 0.3123

Treatment 7c_1st -1.1638 1.1590 1312 -1.00 0.3155

Treatment 7d_1st -1.5975 1.1430 1312 -1.40 0.1624

Treatment 9a_1st 1.8643 1.1354 1312 1.64 0.1009

Treatment 9b_1st 1.5452 1.1749 1312 1.32 0.1887

Treatment 9c_1st -1.1694 1.1078 1312 -1.06 0.2913

Treatment 9d_1st -1.8980 1.2651 1312 -1.50 0.1338

yearmean*Treatment 11a_1st 1.0153 0.2084 1312 4.87 <.0001

yearmean*Treatment 11b_1st 0.8780 0.1741 1312 5.04 <.0001

yearmean*Treatment 11c_1st 1.0929 0.1989 1312 5.49 <.0001

yearmean*Treatment 11d_1st 1.3568 0.2481 1312 5.47 <.0001

yearmean*Treatment 13a_1st 1.1065 0.2114 1312 5.23 <.0001

yearmean*Treatment 13b_1st 1.1897 0.2205 1312 5.40 <.0001

yearmean*Treatment 13c_1st 1.2013 0.2038 1312 5.90 <.0001

yearmean*Treatment 13d_1st 1.0936 0.2089 1312 5.24 <.0001

yearmean*Treatment 17a_1st 0.8566 0.1483 1312 5.78 <.0001

yearmean*Treatment 17b_1st 0.7749 0.1451 1312 5.34 <.0001

yearmean*Treatment 17c_1st 0.8666 0.1651 1312 5.25 <.0001

yearmean*Treatment 17d_1st 0.6195 0.1618 1312 3.83 0.0001

yearmean*Treatment 3a_1st 0.7037 0.1444 1312 4.87 <.0001

yearmean*Treatment 3b_1st 0.7573 0.1428 1312 5.30 <.0001

yearmean*Treatment 3c_1st 0.6929 0.1530 1312 4.53 <.0001

yearmean*Treatment 3d_1st 0.8804 0.1606 1312 5.48 <.0001

yearmean*Treatment 6a_1st 1.1765 0.1721 1312 6.83 <.0001

yearmean*Treatment 6b_1st 1.0743 0.1756 1312 6.12 <.0001

yearmean*Treatment 7a_1st 1.1838 0.1681 1312 7.04 <.0001

yearmean*Treatment 7b_1st 0.9871 0.1717 1312 5.75 <.0001

yearmean*Treatment 7c_1st 1.1652 0.1768 1312 6.59 <.0001

yearmean*Treatment 7d_1st 1.0168 0.1742 1312 5.84 <.0001

yearmean*Treatment 9a_1st 0.9329 0.1730 1312 5.39 <.0001

yearmean*Treatment 9b_1st 0.9807 0.1794 1312 5.47 <.0001

yearmean*Treatment 9c_1st 1.2107 0.1684 1312 7.19 <.0001

yearmean*Treatment 9d_1st 1.1860 0.1941 1312 6.11 <.0001

**Test of heterogeneity of slopes:**

Type III Tests of Fixed Effects

Effect DF DF F Value Pr > F

Treatment 25 1312 2.85 <.0001

yearmean 1 1312 59.26 <.0001

yearmean*Treatment 25 1312 3.17 <.0001

The interaction is highly significant (F = 3.17, p < 0.001) according to a Wald-type F-test, showing that the slopes are significantly different between treatments.

***References***

1. Denis, J.B. (1988) Two way analysis using covariates. Statistics 19:123-132
2. Digby, P. G. N. (1979). Modified joint regression analysis for incomplete variety x environment data. *Journal* of *Agricultural Science, Cambridge* 93, 81-86.
3. Eberhart, S. A., and Russell, W.A. (1966) Stability parameters for comparing varieties. Crop Sci, 6, 36-40.
4. Finlay KW, Wilkinson GN (1963) The analysis of adaptation in a plant-breeding programme. Aust J Agric Res 14:742-754
5. Gabriel, K.R., and Zamir, S. (1979), “Lower rank approximation of matrices by least squares with any choice of weights,”*Technometrics*, 21, 489–498.
6. Guo X, Dutta S, Nettleton D 2021 A hierarchical spatial Finlay-Wilkinson model for analysis of multi-environment field trials. Talk presented at AgStat conference 2021, Gainesville, Florida.
7. Li X, Guo T, Mu Q, Li X, Yu J 2018 Genomic and environmental determinants and their interplay underlying phenotypic plasticity. Proceedings of the National Academy of Science 115, 6679-6684.
8. Nabugoomu F, Kempton RA, Talbot M 1999 Analysis of series of trials where varieties differ in sensitivity to locations. Journal of Agricultural, Biological, and Environmental Statistics 4, 310-325.
9. Ng MP and Grunwald GK 1997 Nonlinear Regression Analysis of the Joint-Regression Model. Biometrics 43, 1366-1372.
10. Ng MP, and Williams ER 2001 Joint‐regression Analysis for Incomplete Two‐way Tables. Australian and New Zealand Journal of Statistics 43, 201-206.
