# Supplementary material for: Long-term trends in yield variance of temperate managed grassland
Source: Agron Sustain Dev. 2023 Apr 26;43(3):37. doi: 10.1007/s13593-023-00885-w (PMC10133363; doi:10.1007/s13593-023-00885-w)
Supplement: Supplementary file 9 — Supplementary file9 (DOCX 73 KB) [file 13593_2023_885_MOESM9_ESM.docx]

 **Fig. A9 Supplementary material** Summary plots for the seven fertilization treatments showing the temporal trends in mean yield (blue dotted line) and yield variance (red bars) each as a mean over all liming treatments (1965-2018): **(a)** Nil; **(b)** PKNaMg; **(c)** N1PKNaMg; **(d)** N*1; **(e)** N2PKNaMg; **(f)** N3PKNaMg; **(g)** FYM/PM. Yield variance denoted Römer`s environmental variance, with lower values indicating more stable yields and higher values indicating more variable yields. Underlying plot specific results are provided in Fig. 4 and an overall overview in Fig. 3. Detailed information about treatments is shown in Table A3 Supplementary material.
