# Supplementary material for: Long-term trends in yield variance of temperate managed grassland
Source: Agron Sustain Dev. 2023 Apr 26;43(3):37. doi: 10.1007/s13593-023-00885-w (PMC10133363; doi:10.1007/s13593-023-00885-w)
Supplement: Supplementary file 10 — Supplementary file10 (DOCX 20 KB) [file 13593_2023_885_MOESM10_ESM.docx]

| **Parameter** | **Mean yield** | | **Yield variability** | |
| --- | --- | --- | --- | --- |
|  | Correlation coefficient | p value | Correlation coefficient | p value |
| Shannon`s diversity index | -0,15 | 0,04 | -0,30 | 0,01 |
| Species number | -0,50 | 0,01 | -0,30 | 0,00 |
| Proportion legumes | -0,18 | 0,06 | -0,20 | 0,05 |

**Table A10 Supplementary material** Correlation across treatments between plant species diversity indices and mean yield, or rather yield variance. Bivariate correlation coefficient: Kendall's tau (τ) and associated p value; analyses based on available years of data/surveys (1991-2000, 2010-2012).
