# Supplementary material for: Long-term trends in yield variance of temperate managed grassland
Source: Agron Sustain Dev. 2023 Apr 26;43(3):37. doi: 10.1007/s13593-023-00885-w (PMC10133363; doi:10.1007/s13593-023-00885-w)
Supplement: Supplementary file 11 — Supplementary file11 (DOCX 152 KB) [file 13593_2023_885_MOESM11_ESM.docx]

**
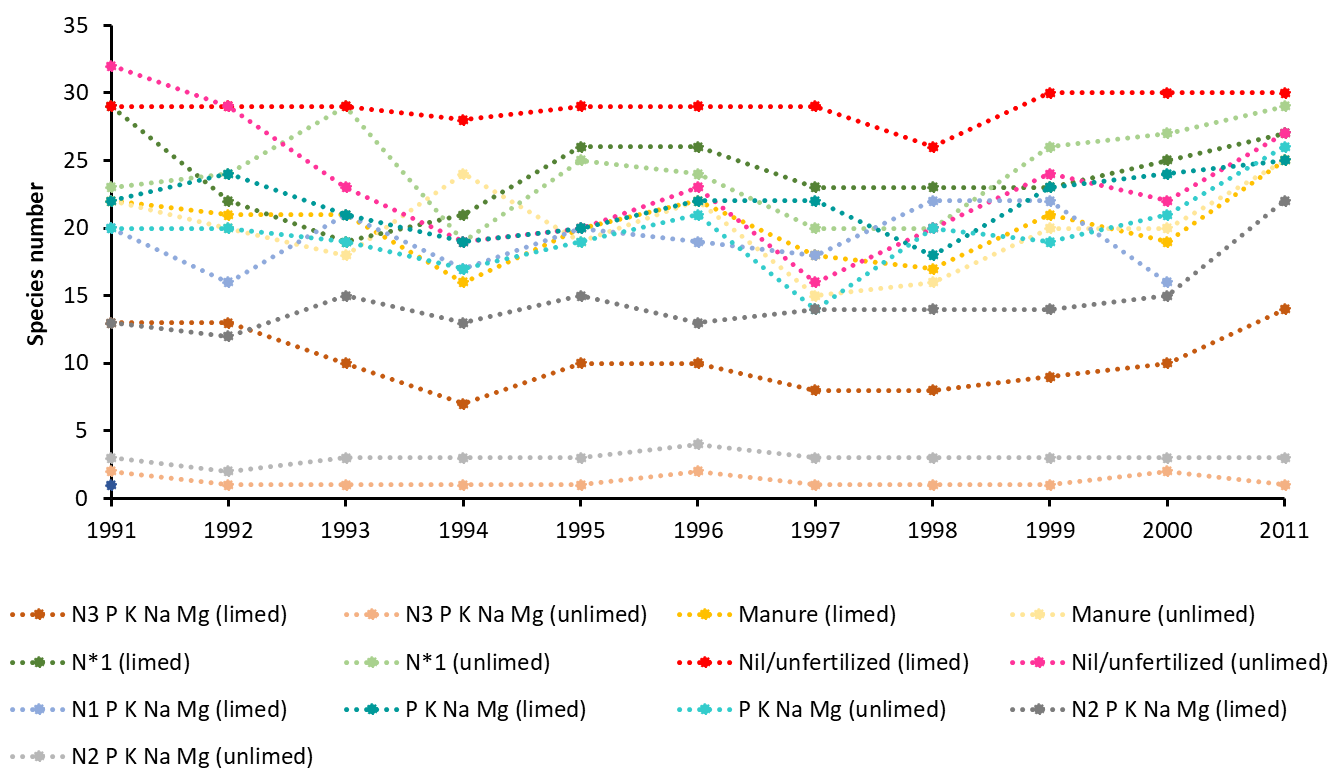


Fig. A11 Supplementary material** Changes in plant species number over time. Number of plant species comprising 1% or more of biomass. Source: Underlying data used for this figure have been made publicly available on the e-RA website (doi: 10.23637/rpg5-species_1991-2000-01) (Perryman et al. 2021).
